# iGlioSub: an integrative transcriptomic and epigenomic classifier for glioblastoma molecular subtypes

**DOI:** 10.1186/s13040-021-00273-8

**Published:** 2021-08-23

**Authors:** Miquel Ensenyat-Mendez, Sandra Íñiguez-Muñoz, Borja Sesé, Diego M. Marzese

**Affiliations:** Cancer Epigenetics Laboratory at the Cancer Cell Biology Group, Institut d’Investigació Sanitària Illes Balears (IdISBa), Carretera de Valldemosa 79, -1F, 07120 Palma de Mallorca, Spain

**Keywords:** Glioblastoma, Machine learning, Molecular subtypes, Epigenetics, iGlioSub, Cancer, Integrative classifier, DNA methylation, Gene expression

## Abstract

**Background:**

Glioblastoma (GBM) is the most aggressive and prevalent primary brain tumor, with a median survival of 15 months. Advancements in multi-omics profiling combined with computational algorithms have unraveled the existence of three GBM molecular subtypes (Classical, Mesenchymal, and Proneural) with clinical relevance. However, due to the costs of high-throughput profiling techniques, GBM molecular subtyping is not currently employed in clinical settings.

**Methods:**

Using Random Forest and Nearest Shrunken Centroid algorithms, we constructed transcriptomic, epigenomic, and integrative GBM subtype-specific classifiers. We included gene expression and DNA methylation (DNAm) profiles from 304 GBM patients profiled in the Cancer Genome Atlas (TCGA), the Human Glioblastoma Cell Culture resource (HGCC), and other publicly available databases.

**Results:**

The **i**ntegrative **Glio**blastoma **Sub**type (iGlioSub) classifier shows better performance (mean AUC = 95.9%) stratifying patients than gene expression (mean AUC = 91.9%) and DNAm-based classifiers (AUC = 93.6%). Also, to expand the understanding of the molecular differences between the GBM subtypes, this study shows that each subtype presents unique DNAm patterns and gene pathway activation.

**Conclusions:**

The iGlioSub classifier provides the basis to design cost-effective strategies to stratify GBM patients in routine pathology laboratories for clinical trials, which will significantly accelerate the discovery of more efficient GBM subtype-specific treatment approaches.

**Supplementary Information:**

The online version contains supplementary material available at 10.1186/s13040-021-00273-8.

## Introduction

Despite aggressive multimodal treatments, the median survival of glioblastoma (GBM) patients is 15 months, with only 5 % survival beyond 5 years [1]. Some factors influencing the resistance to treatments include sub-optimal drug selection, intra-tumor heterogeneity, tumor genetic background, and epigenetic alterations [2–5]. Epigenetic mechanisms involving DNA methylation (DNAm) and histone modifications play a significant role in cancer progression and resistance [6, 7]. Currently, GBM molecular features are gaining more attention, given their critical role in clinical decision-making. For example, tumors harboring mutations on the *IDH1* gene showed an increase in global DNAm levels, defined as the glioma CpG island Methylator Phenotype (G-CIMP), associated with a better prognosis [8–10]. Additionally, hypermethylation of the *MGMT* gene promoter region is the best-known prognostic factor for a favorable response to Temozolomide [11]. However, other factors such as ZNF7 expression levels, or transcriptional profiling of the tumor microenvironment, have emerged as potential prognostic markers for GBM [12, 13].

Originally, GBM was classified into four major molecular subtypes: i.e., Classical, Mesenchymal, Neural, and Proneural [14]. However, in the last years, different studies have shown that the Neural subtype may be a consequence of contamination by oligodendrocytes and neurons from tumor margins instead of being characteristic of the tumor itself [15]. Identification of GBM subtypes is essential for prognosis, as they have different clinical outcomes and molecular characteristics [3]. Nevertheless, while the anatomic-pathologic diagnosis of GBM is a well-established routine procedure that aids oncologists in deciding clinical management, an accurate molecular stratification of GBM still requires high-throughput molecular profiling, which is expensive, requires extensive data processing, and is not routinely available on regular health care centers.

Increased accessibility to multi-omic profiles of clinically annotated cancer specimens has accelerated the identification of molecular classification systems [16, 17]. To date, several classifiers based on gene expression or DNAm features have been generated to guide the treatments of multiple cancer types [14, 18]. However, most of these need to perform whole-transcriptome or epigenome-wide profiling, requiring technology that is not accessible in all health centers. To override this limitation, we and others have constructed classifiers using data reduction techniques that provide the minimum and highly informative number of features necessary for stratifying cancer specimens [19–22]. For instance, these methodologies have already been used to construct various gene expression-based panels, including a 44-gene panel to classify renal cell carcinomas [23] or a 13-gene panel to estimate radiation sensitivity in the head and neck squamous cell carcinomas [24]. DNAm-based panels have also shown utility for clinically relevant cancer stratification [25, 26]. For example, identification of the tissue-of-origin in cancer of unknown primary tissue [25], determining the diagnosis of primary tumors affecting the central nervous system [27], and discerning primary from metastatic brain tumors [28, 29]. As a result, these molecular classifiers with a minimum number of features have emerged as a cost-effective alternative that requires low complexity techniques, such as qPCR, methylation-specific PCR, pyrosequencing, etc., that are usually available in pathology laboratories [19, 22].

To contribute to evaluating GBM subtypes in clinical settings, we aimed to construct classifiers that can be adapted to routinely used screening assays. We have employed machine learning, computational biology algorithms, and prioritization of highly informative transcriptomic and epigenomic features to construct and validate three types of classifiers: (1) a gene expression-based classifier, (2) a DNAm-based classifier, and (3) a novel **Glio**blastoma **Sub**type classifier (hereinafter referred as iGlioSub) containing both features.

## Methodology

### Data access, collection, and normalization

The Cancer Genome Atlas (TCGA) clinical data [2], containing annotations from 1,122 glioma patients, was obtained from the Broad Institute Genome Data Analysis Center (GDAC) Firehose [30] on 20/2/2019. This data was curated, excluding low-grade glioma (LGG) cases or incomplete clinical and demographic information, such as age, gender, Karnofsky Performance Score, tumor purity, subtype information, or IDH mutation status, to reduce the variability between the groups and avoid confounding factors (Suppl. Figure 1). These patients were classified into Classical, Mesenchymal, and Proneural subtypes using the “Transcriptome subtype” or the “Original Subtype” annotation. The agreement between these two annotations, representing transcriptome-based and immunohistochemistry (IHC)-based stratification of patients, was computed using the kappa coefficient using the R/*psych.* Due to the conflicting evidence, all cases classified as Neural were excluded from downstream analyses [15, 31]. The resulting cohort included 238 GBM patients (Suppl. Figure 1). Gene expression data (Affymetrix u133a array data (“ht_hg_u133a-gene_rma (MD5)”) was downloaded from Firehose Broad on 5/3/2019. In addition, gene expression data (u133a microarray) from 44 established primary GBM cultures with previously established molecular subtypes were downloaded from the Human Glioblastoma Cell Culture resource (HGCC) [32]. DNAm data was downloaded from Genomic Data Commons (GDC) using *R/TCGAbiolinks* [33] on 11/3/2019 using R 4.0.2 and Bioconductor 3.11. After filtering missing probes, DNAm data generated with the HumanMethylation27 (HM27K; *n* = 133 patients) and the Infinium HumanMethylation450 (HM450K; *n* = 44 patients) BeadChip arrays were combined and included in the study. Probes common to all Illumina BeadChip generations and passing the GenomeStudio QC (Illumina; *n* = 22,330 probes) were employed as input for the downstream analyses. Furthermore, HM450K DNAm data from 35 additional patients were downloaded from the NCBI-GEO repository (GSE128654 [34]). Principal Component Analysis (PCA) was performed using the *prcomp* function on R to identify potential batch effects in DNAm and gene expression datasets.

### Data processing and statistical analysis

For the gene expression analysis, this process was performed using the training cohort from TCGA (*n* = 234). First, the mean expression of all genes was computed, and the 25 % of genes with the lowest overall expression levels were removed from the dataset (*n* = 3,011). The expression values of the remaining genes (*n* = 9,031) were normalized to Z-Score using the ‘*scale’* command in R. As a general analytical scheme, the differences between ‘Subtype’ and ‘Control’ (all subtypes except the selected one) were computed by Z-Ratio. The Student’s t-test was employed to evaluate the statistical significance of differences between Z-Scores. The obtained *p*-values were corrected for multiple comparisons (*q*-value) by the “False discovery rate” (FDR) method using the command *p.adjust* in R. The differences between the groups were considered significant when the absolute Z-Ratio was over 1.5, with *q*-value < 0.05. All significantly differentially expressed genes (DEG) were selected for downstream analyses. Heatmaps were employed to visualize hierarchical cluster analyses based on Euclidean distance using R/*gplots*, and t-distributed Stochastic Neighbor Embedding (t-SNE) was performed to identify the data’s distribution using R/*Rtsne* (perplexity = 30, maximum iterations = 5,000, theta = 0).

**Fig. 1:**
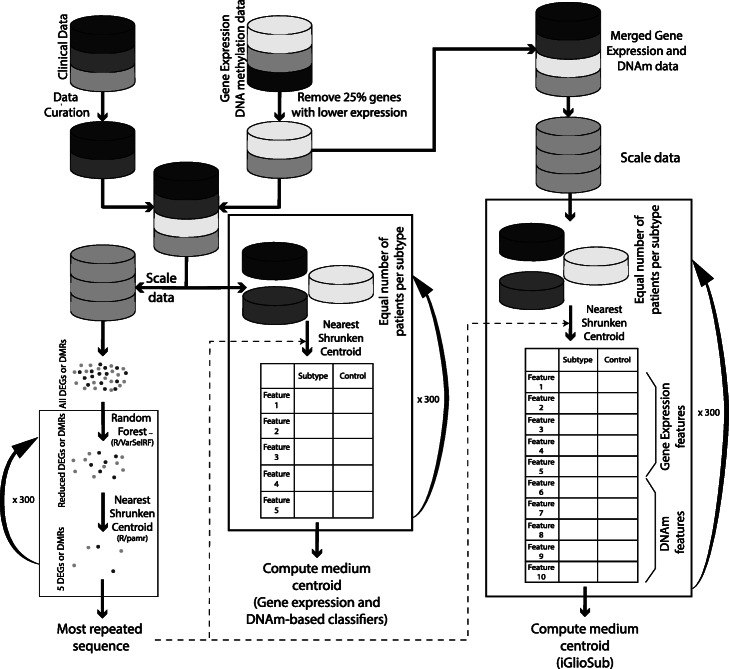
Schematic representation of the methodology for obtaining the gene expression-based, DNAm-based, and iGlioSub panels to classify GBM subtypes

For the analysis of the DNAm data, we merged three databases (TCGA-HM27K, TCGA-HM450K, and GSE128654 -HM450K-) to construct and validate DNAm-based classifiers. The batch effect associated with the different data sources was identified by PCA and corrected using the *ComBat* function on the R/*sva* package (Suppl. Figure 2). The DNAm levels were converted to M-value to transform the data from a bimodal to a normal distribution [35]. The differentially methylated sites (DMS) were identified in a training cohort (70 %; see Suppl. Table 1). Genomic regions with differences in M-value between subtype and control of at least 1.0 and *q*-value < 0.05 were considered DMS (Fig. 1).

### Generation of machine learning-based classifiers

First, the initial number of features was reduced by Random Forest (RF) algorithms applying the varSelRF command from R/*varSelRF* package to all the DEG or DMS (varSelRF parameters: mtryfactor = 1, ntre = 1000, ntreeIterat = 500, vars.drop.frac = 0.2). This method uses RF and, in each iteration, discards the 20 % of features with the lowest relevance in stratifying the patients, and, finally, selects the combination of features with the lowest Out-Of-Bag (OOB) error, the variable with the highest importance in the selection of the best combination of features [36, 37]. Then, a classification system using the nearest shrunken centroid (NSC) method was created, according to the potential of each feature to discriminate the subtype and control groups. The R/*pamr* package was employed for the elaboration of this system. 234 patients were included in the training phase for the gene expression classifier and 126 patients for the DNAm classifier. A 10-fold cross-validation strategy was employed to test each classifier. The top five highly ranked features meeting the required conditions were selected using the *pamr.listgenes* command. These five features mandatorily included at least two positively and two negatively associated features for the specific subtype. This process, beginning with R/*varSelRF*, was repeated 300 times for each subtype, and then the most repeated sequence of genes was chosen.

Sub-cohorts containing the same number of patients per subtype were created randomly selecting cases from the initial set of patients to evaluate centroids’ assignment. For the gene expression data, the data was re-normalized for this new cohort using the ‘*scale’* command in R. The centroids for the selected features were calculated using R/*pamr*. This process was repeated 300 times with different randomly selected subtype-balanced cohorts. In each iteration, we calculated the centroids, the standard error of each centroid, and the error rate of the panel. The efficiency of the final centroids was assessed by establishing the Receiver Operating Curve (ROC) and calculating the Area Under the Curve (AUC) using the R/*ROCR* package in each iteration. Finally, the mean of all iterations was calculated for all parameters. Error rates and AUC differences between gene expression, DNAm, and integrative panels were independently analyzed in each subtype by ANOVA (Tukey’s multiple comparison test for individual comparison between panels) using R commands *aov* and *TukeyHSD* (Fig. 1).

HGCC gene expression data (*n* = 44) was used as an independent validation cohort for the gene expression-based classifier, and the performance of the DNAm classifier was assessed in a validation cohort (30 %, *n* = 54; see Suppl. Table 1). The centroids generated in our study were used to obtain scores for each GBM subtype and patient, and the subtype with the highest score in each sample was selected. Agreement between our subtype assignment and the preset classification was evaluated using the kappa coefficient using the R/*psych*.

### Generation of integrative classifiers

The informative features from the gene expression and DNAm final classifiers were used to construct the integrative classifier. The integrative classifier centroids were generated as described for gene expression- and DNAm-based panels classifiers, but skipping the step of feature selection. The efficiency was assessed in a validation cohort (30 %, *n* = 49; see Suppl. Table 1).

### Assessment of tumor subtype heterogeneity and potential impact in classification performance

Single-cell RNA-seq (scRNA-seq) data from 25 GBM cases [4] was downloaded from BROAD Institute Single Cell Portal. All cells (*n* = 7,860) were individually classified using GlioVis, which was previously proved to classify GBM cells using scRNA-seq data [38, 39], and our gene expression-based classifiers. The percentage of cells classified as Classical, Mesenchymal, and Proneural established by GlioVis and our methods was estimated for each patient. The mean expression of each gene was estimated for each GBM patient, and the resulting gene expression profiles were classified using GlioVis and the gene expression classifier.

### Gene enrichment analysis and identification of potential druggable targets

The Metascape data analysis resource [40] was used to evaluate gene ontology and gene pathway enrichment analyses. The data was loaded as a Multiple Gene List with configuration to obtain Gene Ontology terms (GO) enrichment. All genes included in the analysis (*n* = 9,031) were used as background genes. We manually curated each GO term to include molecular functions relevant for GBM progression. The selected GO terms were represented using the R/*ggplot2* package. A subset of clinically relevant pathways was selected and evaluated using Gene2Drug [41] to obtain a list of drugs potentially targeting the subtype-specific pathways.

### Identification of association between DMS and gene regulatory elements

The Genomic Regions Enrichment of Annotations Tool (GREAT) [42] was employed to identify associations between DMS and gene regulatory elements for each GBM subtype. Briefly, two Browser Extensible Data (BED) files per subtype containing hypermethylated and hypomethylated DMS were evaluated using all the genomic regions used in the initial analysis as background (*n* = 22,330). Genes located up to 5 kb upstream or downstream of the genomic coordinate of the DMS were considered proximal, and genes up to 1 Mb were considered distal. The most relevant pathways associated with GBM were plotted using R/*ggplot2*.

## Results

### Machine learning-based transcriptomic and epigenomic classifiers efficiently classify GBM subtypes

DEG and DMS were estimated by comparing the transcriptomic or epigenomic profiles of each subtype with the rest of the samples. Unsupervised hierarchical clustering analysis and t-SNE representation using subtype-specific DEG or DMS showed a modest overall performance in segregating the cases according to the annotated GBM subtype, even when combining DEG and DMS (Suppl. Figures 3 and 4). We, therefore, employed RF to identify informative genomic features amongst DEG and DMS to stratify GBM specimens into molecular subtypes. The initial RF-based signatures were reduced by applying NSC approaches. This additional step allowed to exclude correlated and possibly redundant features to generate signatures with a minimum number of genes or CpG sites. We identified that five features per subtype were the minimum number of features with high accuracy and low error rates (Suppl. Figure 5). Each classifier included features positively and negatively associated with each subtype to construct gene expression (Suppl. Table 2), DNAm (Suppl. Table 3), and integrative (iGlioSub; Suppl. Table 4) classifiers.

**Fig. 2:**
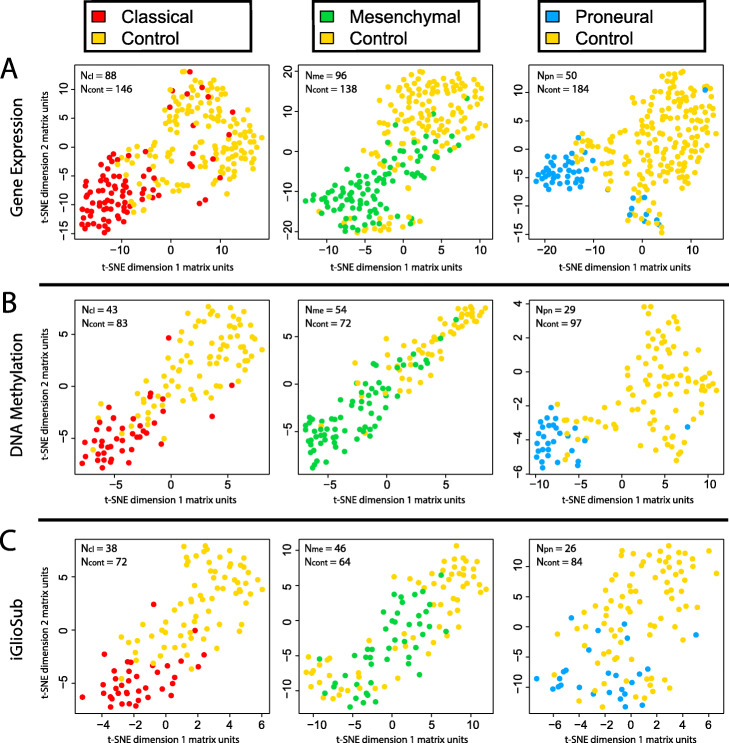
t-SNE plots representing the clustering of GBM specimens using (**A**) the 5 genes included in each gene expression-based panel, **B** the five CpG sites included in each DNAm-based panel, and **C** the ten features included in each iGlioSub panel. All plots show a significant subtype-specific patient stratification

**Fig. 3:**
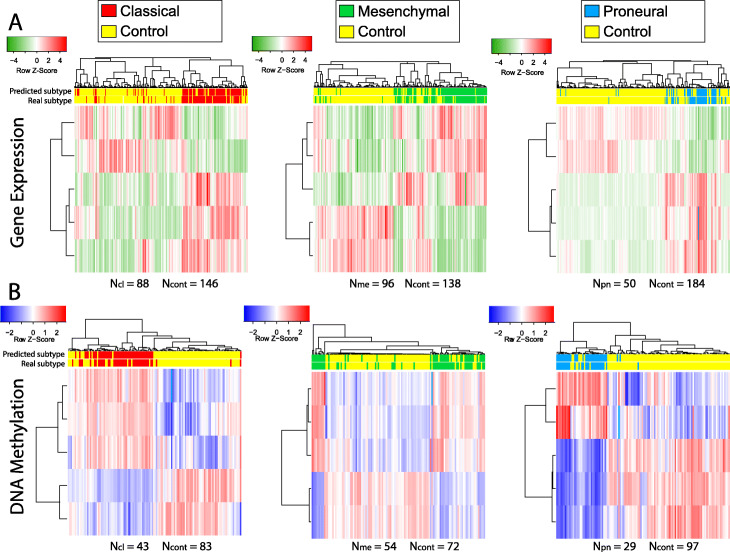
Hierarchical cluster analysis using Euclidean distance using (**A**) 5 genes included in each gene expression-based panels or (**B**) 5 CpG sites included in DNAm-based panels for each GBM subtype. All plots show a significant classification potential of hierarchical clustering

The machine learning-based classifiers improved the stratification of GBM cases according to the annotated subtype compared to the DEG and DMS (Figs. 2 and 3). Each classifier was designed to stratify a specific GBM subtype (Classical, Mesenchymal, or Proneural) from the rest. Thus, combining all the features into a unified classifier, independently of the data type, had a worse performance than stratifying GBM specimens using subtype-specific classifiers (Suppl. Figures 6 and 7).

### iGlioSub is more effective in stratifying specimens into GBM subtypes than the DNAm- and gene expression-based classifiers

**Fig. 4:**
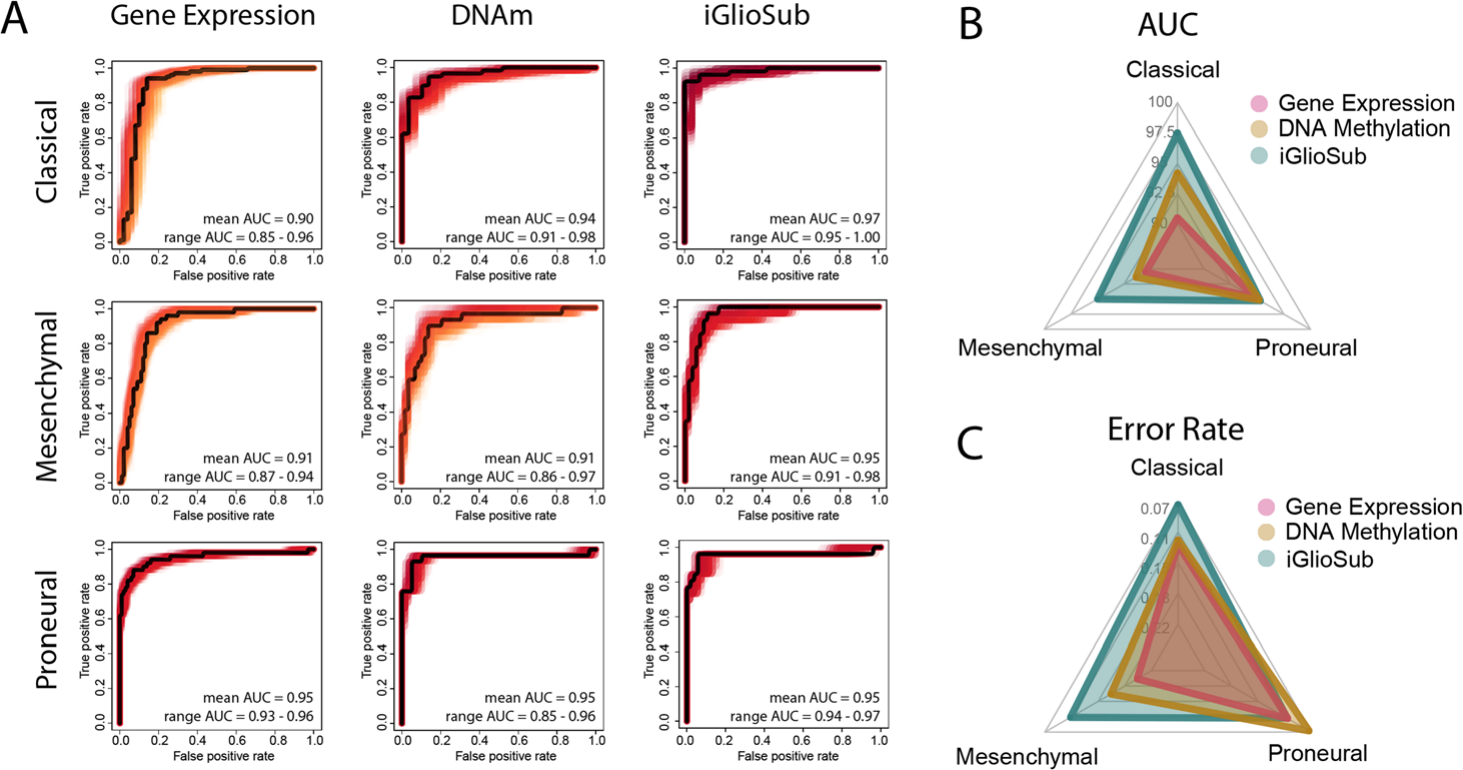
**A** ROC Curves for the gene expression, DNAm, and iGlioSub panels for the Classical, Mesenchymal, and Proneural subtypes. A black line represents a representative ROC Curve of each panel. **B-C** Radar plot representing the (**B**) AUC and (**C**) Error Rate of the gene expression, DNAm, and iGlioSub panels for the three GBM subtypes. iGlioSub displays a better performance in both AUC and Error Rate than DNAm and gene expression-based panels for the Classical and Mesenchymal subtypes (*p* < 0.01) but not for the Proneural subtype

The predictive performance of the classifiers was compared by establishing the ROC curves and calculating the AUC (Fig. 4A). Our analysis showed that iGlioSub had higher AUC to identify the Classical (97.5 ± 1.0 %) and the Mesenchymal (95.0 ± 1.3 %) subtypes compared to the gene expression (90.5 ± 2.1 %, 90.5 ± 1.3 %) and DNAm (94.2 ± 1.4 %, 91.4 ± 2.1 %) classifiers (*p* < 0.01). The iGlioSub Proneural panel (95.3 ± 0.5) displayed better performance than the gene expression classifier (94.8 ± 0.6 %; *p* < 0.01) but was not significantly better than the DNAm classifier (95.2 ± 0.5 %; *p* > 0.05; Fig. 4B). The error rate was also inferior in the iGlioSub panel for the Classical (0.07 ± 0.019) and the Mesenchymal (0.11 ± 0.021) subtypes compared to the gene expression (0.12 ± 0.013, 0.20 ± 0.024; *p* < 0.01) and the DNAm panels (0.12 ± 0.020, 0.16 ± 0.024; *p* < 0.01). Similar to the AUC, the iGlioSub panel for the Proneural subtype displayed a significantly higher error rate (0.10 ± 0.020) than the DNAm-based classifier (0.07 ± 0.017, *p* < 0.01), but no different to the gene expression classifier (0.10 ± 0.012, *p* > 0.05; Fig. 4C).

An external cohort of 44 tumors from the HGCC resource was used to validate the gene expression-based panels. Prediction of GBM subtypes in these samples showed a moderate agreement (κ = 0.68; *p* < 0.01), thus exhibiting an overall high accuracy of our gene expression-based panel. The agreement was slightly variable in different GBM subtypes, ranging from 0.60 for the Classical subtype and 0.68 for the Mesenchymal subtype to 0.78 for the Proneural subtype (Suppl. Tables 5 and 6).

In a validation cohort (*n* = 54 cases), the DNAm-based classifier exhibited a higher agreement than the gene expression-based classifier (κ = 0.82, *p* < 0.01). This was observed for every subtype (Classical = 0.84; Mesenchymal = 0.77; Proneural = 0.85; Suppl. Tables 7 and 8).

The iGlioSub efficiency was assessed in a validation cohort (*n* = 49). The agreement of this classifier was superior to the DNAm-based and the gene expression-based classifiers (κ = 0.9, *p* < 0.01), and consistent in all subtypes (Classical = 0.87; Mesenchymal = 0.88; Proneural = 1; Suppl. Tables 9 and 10).

### Machine learning transcriptomic classifiers identify GBM subtype heterogeneity

scRNA-seq data from 25 cases were used to assess the capacity of the machine learning transcriptomic classifier to identify subtype-heterogeneity in GBM. All the cells (*n* = 7,860) were initially classified using the GlioVis method [39]. In line with prior observations [43], all the cases presented a variable proportion of the GBM subtypes (Suppl. Figure 8). Twenty-one out of twenty-five patients showed a prevalent subtype (> 50 % of all the cells) using the transcriptomic classifier. Importantly, our gene expression classifier showed similar results in the classification of each GBM cell and the proportion of each subtype per sample (*p*-value < 0.01; Suppl. Figure 8). To evaluate how the GBM heterogeneity may impact the identification of GBM subtypes, we simulated bulk RNA-sequencing (RNA-seq) profiling for each of the 25 cases by establishing the mean value for each gene. We found that GlioVis identified the predominant GBM subtype in 19 out of 25 cases (κ = 0.63) and our classifier in 23 out of 25 (κ = 0.88; Suppl. Figure 8).

### Differential activation of immune-related pathways among GBM subtypes

**Fig. 5:**
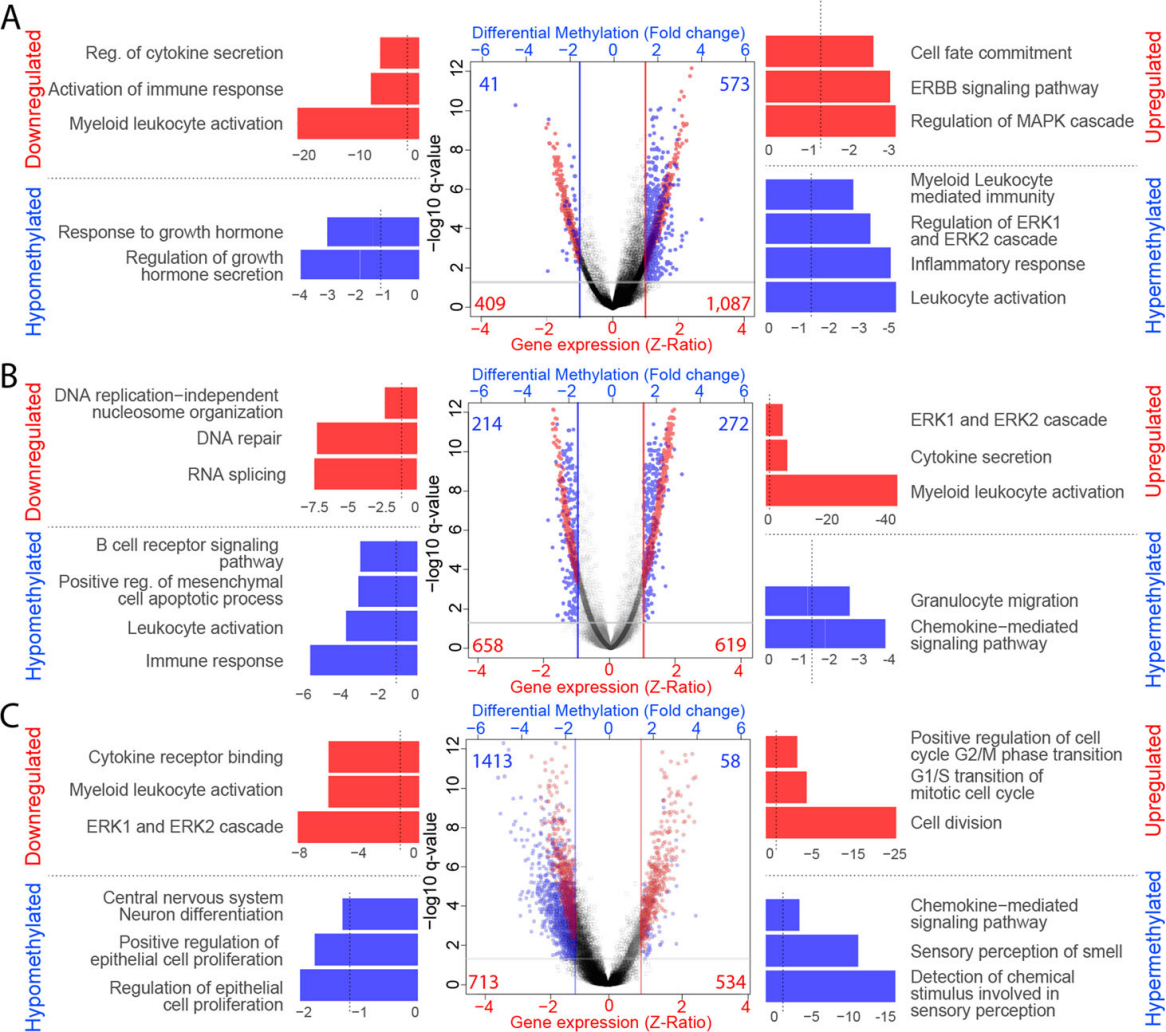
Analysis of differentially expressed genes and differentially methylated sites in GBM Subtypes. Left panel: Gene Enrichment Analysis of downregulated genes (red bars) and hypomethylated CpG sites (blue bars) in each subtype. Vertical lines represent the *p*-value cutoff (*p* = 0.05). Middle panel: Volcano Plot, representing differential gene expression (Z-Ratio; red dots) and differentially methylated CpG sites (M-value fold change; blue dots). Horizontal lines indicate the *q*-value threshold (Student’s t-test, corrected by FDR = 0.05). Right panel: Gene Enrichment Analysis of upregulated genes (red bars) and hypermethylated CpG sites (blue bars) in each subtype, being (**A**) Classical subtype, **B** Mesenchymal subtype, and **C** Proneural subtype. Vertical lines represent the *p*-value cutoff  (*p* = 0.05)

The classical subtype showed enrichment in upregulated genes in the ErbB receptor tyrosine kinase family signaling pathway and cell fate commitment, among other pathways. In contrast, the downregulated genes were enriched in immune system-related pathways, such as regulation of cytokine secretion and myeloid leukocyte activation (Fig. 5A, Suppl. Figure 9). Furthermore, many hypermethylated sites were involved in immunity pathways, such as leukocyte migration and activation, supporting the observation from the gene expression analysis (Fig. 5A, Suppl. Figure 10).

The patients with the Mesenchymal subtype showed higher expression of genes in the ERK1/2 cascade and pro-inflammatory, cytokine secretion, and myeloid leukocyte activation pathways. On the other hand, downregulated genes were enriched in RNA splicing pathways and DNA repair genes (Fig. 5B, Suppl. Figure 9). In these patients, the hypermethylated sites were enriched in genes in chemokine-mediated signaling and granulocyte migration pathways, while hypomethylated sites were enriched in immune response, regulation of immune system process, and leukocyte activation pathways (Fig. 5B, Suppl. Figure 10).

Finally, the Proneural subtype showed upregulated genes enriched in cell division pathways, including G1/S and G2/M transition, and downregulated genes enriched in inflammatory pathways, such as myeloid leukocyte activation and cytokine receptor binding, and ERK1/2 cascade (Fig. 5C, Suppl. Figure 9). Surprisingly, only 58 sites (4 %) were hypermethylated in Proneural samples, which involved varied pathways such as central nervous system neuron differentiation. The hypermethylated sites were enriched in pathways related to chemokine-mediated signaling pathways and leukocyte migration, matching the gene expression results, which showed downregulation of these pathways (Fig. 5C, Suppl. Figure 10).

Using the ‘Gene2Drug’ algorithm, several drugs were identified to target enriched pathways in each subtype. For instance, azacitidine and quercetin, two FDA-approved drugs, diminish the ERBB signaling pathway, a hallmark of the Classical subtype. Furthermore, erastin, a drug tested as a sensitizer for TMZ in GBM [44], reduces the levels of genes in the ERK1/2 cascade, which are typically upregulated in the Mesenchymal subtype. Flupentixol, an antipsychotic drug used by schizophrenia patients, diminishes genes related to G1/S and G2/M transition, specifically upregulated in the Proneural subtype. Furthermore, this drug has been proposed as a novel anticancer therapy in lung cancer [45].

## Discussion

Even though GBM subtypes were first described more than a decade ago [14], there are still no subtype-specific treatments. This is partly due to the lack of systematic classification methods that can be routinely employed in clinical laboratories. Current approaches based on transcriptome or genome-wide DNAm profiling have high costs and complexity [46]. Thus, a simplified and cost-effective classification methodology could overcome this limitation and allow neuro-oncologists to assess subtype-specific treatment efficiency.

Our study provides alternatives to the current classification methodologies by reducing the number of required informative features. These classifiers based on gene expression, DNAm, or an integration of these data types, provide similar performances to the current classification. The main advantage of these novel classifiers is the potential adaptability to low complexity techniques such as qPCR or pyrosequencing, as we have previously shown to identify primary and metastatic brain tumors [28]. In fact, pyrosequencing is already being routinely used to determine DNAm levels of the *MGMT* gene promoter region, one of the most important prognostic and predictive factors in GBM [47].

Importantly, we showed that iGlioSub reaches a classification efficiency, assessed by the kappa agreement coefficient, similar to the TCGA IHC-based classification [48] and superior to other machine learning-based strategies for the classification of GBM subtypes (accuracy = 0.9) [49]. This efficiency is also comparable to classifiers applied in other tumors (accuracy = 0.9–0.95) [23, 28]. This excellent performance of the reduced classifiers was achieved by consecutively combining two machine learning approaches, RF and NSC, to identify robust minimum feature signatures. Of note, we found that using as little as five features per molecular subtype in the final classifier showed an optimal performance. Furthermore, we identified that the gene expression-based classifier could successfully identify subtype-heterogeneity in scRNA-seq data, suggesting that our classifiers identify the predominant GBM molecular subtype in each patient. Unfortunately, due to the lack of single-cell DNAm datasets, the iGlioSub and DNAm-based classifiers could not be evaluated.

Interestingly, we found a significant overlap between our classifiers and Wang’s 150-genes signatures (Exact hypergeometric test = 47, *p* < 0.001) [15]. Six out of 15 genes in our classification signatures were present in Wang’s 150-genes signatures (GPR17, SLC1A1, ARPC1B, CTSC, VAV3, and FGFR3), suggesting a potential functional association of these genes with the GBM subtypes. While both gene expression and DNAm-based classifiers have demonstrated a great potential for GBM subtype classification, the DNAm-based classifier exhibited a higher performance with significantly lower error rates. These results are consistent with previous studies demonstrating the auspicious classification potential of DNAm signatures for the molecular classification of tumor samples [25, 28, 50, 51]. Furthermore, iGlioSub, which contains gene expression and DNAm features, outperformed individual methods, with the lowest error rate and the highest AUC in the Classical and Mesenchymal subtypes classification and the best accuracy the classification of an external cohort. Due to the strategy used in this study, the panels perform better when used to discriminate between one subtype and the rest. When we combine the three subtype-specific panels into one single classifier, the performance of the classifier worsens. Nevertheless, in this scenario, iGlioSub outperformed the gene expression and the DNAm combined classifiers. These findings suggest that integrative classifiers using multi-omic data could become a powerful tool for future molecular diagnostic applications in GBM and potentially in other tumors.

Our study also provides insights into the subtype-specific molecular alterations that could be targeted by selective treatments to settle the bases for novel experimental treatments. For example, patients with GBM Mesenchymal subtype present activation of pathways associated with immunity and inflammation processes, such as the production of cytokines or myeloid leukocyte activation. Our DNAm analysis revealed that genomic regions associated with immune-related pathways remained hypomethylated in the GBM with Mesenchymal subtype, suggesting an epigenetic regulation of these traits. These observations agree with recent evidence showing that the Mesenchymal subtype presents a higher immune activity than Proneural and Classical subtypes [52]. Overall, these findings could be particularly relevant to contextualize the clinical observations of GBM patients undergoing immunotherapy [53, 54]. Based on *in silico* analysis, we were able to identify several drug candidates that target different active pathways, which are specifically upregulated in each subtype. Further analysis in this matter would help elucidate the potential applications of these drugs, either individually or in combination with immunotherapy or chemotherapy.

## Conclusions

This study provides three novel classification methods for GBM molecular subtypes, one based on gene expression, a second based on DNAm, and a system that integrates both types of feature, called iGlioSub. Given the ease of using our molecular classifiers, these classifiers could be easily adapted to most clinical laboratories to stratify GBM patients routinely. In addition, this initiative might facilitate a significant data collection that could lead to personalized GBM subtypes treatments and improve the clinical management of this disease.

## Supplementary Information

**Additional file 1: Suppl Figure 1.** Process of curation of the TCGA-downloaded samples.

**Additional file 2: Suppl Figure 2.** t-SNE plots displaying the distribution of samples coming from different DNA methylation arrays and sources, before and after correction of batch effect. The batch effect was corrected using the *ComBat* function in the R/*sva* package.

**Additional file 3: Suppl Figure 3.** t-SNE plotting representing clustering of different subtype patients using (A) all differentially expressed genes (DEG), (B) all differentially methylated sites (DMS), or (C) all DEG and DMS. All plots show a modest classification potential using gene expression data of all DEG, DNAm data of all DMS, or both all DEG and DMS combined.

**Additional file 4: Suppl Figure 4.** Hierarchical cluster analysis using Euclidean distance for the gene expression/DNAm levels of (A) all differentially expressed genes (DEG), (B) all differentially methylated sites (DMS), or (C) all DEG and DMS. All plots show a significant classification potential of hierarchical clustering using gene expression data of all DEG, DNAm data of all DMS, or both all DEG and DMS combined.

**Additional file 5: Suppl Figure 5.** Comparison of the diminution of the Area Under Curve (AUC) (top panels) and increase of the Error Rate (bottom panels) with less than five features per panel for the gene expression (left) and DNAm (right) panels. The lines represent the mean AUC±SD and Error Rate±SD for each subtype. All lines are normalized against the range of the classifier with the highest AUC or the lowest error rate (Classical subtype classifier). Both AUC and Error Rate show an optimal performance when using five features per panel.

**Additional file 6: Suppl Figure 6.** t-SNE plots representing clustering of different subtypes using the combination of the three-subtype panels using gene expression, DNAm, and iGlioSub.

**Additional file 7: Suppl Figure 7.** Hierarchical cluster analysis using Euclidean distance for the gene expression/DNAm levels of the three-subtype panels simultaneously (*n *= 15 features).

**Additional file 8: Suppl Figure 8.** Barplots displaying the percentage of cells classified as Classical, Mesenchymal, and Proneural from a single-cell RNA-seq experiment using our gene expression-based classifier and GlioVis. The colored squares represent the subtype selected by the classifier and GlioVis for each patient using a bulk simulation using the mean expression of all cells. Both methodologies display a moderate agreement in the selection of the predominant subtype (κ=0.45), and a significant agreement between the predominant subtype and the assigned subtype using the bulk-like method (classifier κ=0.88; GlioVis κ=0.63).

**Additional file 9: Suppl Figure 9.** Pathways enriched for upregulated and downregulated genes in Classical, Mesenchymal, and Proneural subtype patients compared to the rest of patients.

**Additional file 10: Suppl Figure 10.** Pathways enriched in genes near hypermethylated or hypomethylated CpG sites in Classical, Mesenchymal, and Proneural subtype patients compared to the rest of patients.

**Additional file 11: Suppl Table 1.** Summary of the cohorts used in each step to create and validate the gene expression-based, DNAm-based, and iGlioSub classifiers.

**Additional file 12: Suppl Table 2.** Medium position of centroids and medium standard deviation for each gene in the gene expression-based panels.

**Additional file 13: Suppl Table 3.** Medium position of centroids and medium standard deviation for each CpG site in the DNAm-based panels.

**Additional file 14: Suppl Table 4.** Medium position of centroids and medium standard deviation for each gene or CpG site in the iGlioSub panels.

**Additional file 15: Suppl Table 5.** Results of applying our gene expression classifier to a validation cohort from the Human Glioblastoma Cell Culture (HGCC).

**Additional file 16: Suppl Table 6.** Confusion matrix of applying our gene expression classifier to a validation cohort from the Human Glioblastoma Cell Culture (HGCC).

**Additional file 17: Suppl Table 7.** Results of applying our DNAm classifier to a validation cohort from TCGA and GSE128654.

**Additional file 18: Suppl Table 8.** Confusion matrix of the application of our DNAm classifier to a validation cohort from TCGA and GSE128654.

**Additional file 19: Suppl Table 9.** Results of the application of the iGlioSub classifier to a validation cohort from TCGA.

**Additional file 20: Suppl Table 10.** Confusion matrix of the application of the iGlioSub classifier to a validation cohort from TCGA.

## Data Availability

All the codes used in this study are available in GitHub: https://github.com/mensenyat/iGlioSub. The datasets included in this study belong to TCGA and can be accessed at the National Cancer Institute (NCI) Genomic Data Commons (GDC) Data Portal https://portal.gdc.cancer.gov, or are accessible through Gene Expression Omnibus (GEO): https://www.ncbi.nlm.nih.gov/geo/ and BROAD Institute Single Cell Portal: https://singlecell.broadinstitute.org/single_cell.

## Abbreviations

AUC: Area Under Curve
DEG: Differentially Expressed Genes
DMS: Differentially Methylated Sites
DNAm: DNA methylation
GBM: Glioblastoma
HGCC: Human Glioblastoma Cell Culture resource
HM27K: Illumina’s Infinium HumanMethylation27 BeadChip
HM450K: Illumina’s Infinium HumanMethylation450 BeadChip
iGlioSub: integrative Glioblastoma Subtype classifier
IHC: Immunohistochemistry
NSC: Nearest Shrunken Centroid
RF: Random Forest
RNA-seq: RNA sequencing
ROC: Receiver Operating Curve
scRNA-seq: Single-cell RNA sequencing
t-SNE: t-Distributed Stochastic Neighbor Embedding
TCGA: The Cancer Genome Atlas
