# Correction to: iGlioSub: an integrative transcriptomic and epigenomic classifier for glioblastoma molecular subtypes

**DOI:** 10.1186/s13040-021-00282-7

**Published:** 2021-11-17

**Authors:** Miquel Ensenyat-Mendez, Sandra Íñiguez-Muñoz, Borja Sesé, Diego M. Marzese

**Affiliations:** Cancer Epigenetics Laboratory at the Cancer Cell Biology Group, Institut d’Investigació Sanitària Illes Balears (IdISBa), Carretera de Valldemosa 79, -1F, 07120 Palma de Mallorca, Spain

**Correction to: BioData Mining 14, 42 (2021)**

**https://doi.org/10.1186/s13040-021-00273-8**

Following publication of the original article [1], the authors identified an error in the Funding section.

The section currently reads:

The research was supported by the Spain Instituto de la Salud Carlos III (ISCIII) grants: Miguel Servet Project (#CP17/00188) and AES 2019 Project (# PI19/01514), the Institut d’Investigació Sanitària Illes Balears (IdISBa) FOLIUM program/Impost turisme sostenible (Govern de les Illes Balears), the Fundación Francisco Cobos, and the Asociación Española Contra el Cáncer (AECC).

The section should read:

The research was supported by the Spain Instituto de la Salud Carlos III (ISCIII) grants: Miguel Servet Project (#CP17/00188) and AES 2019 (#PI19/01514) co-funded by ERDF “A way to make Europe”, the Institut d’Investigació Sanitària Illes Balears (IdISBa) FOLIUM program/Impost turisme sostenible (Govern de les Illes Balears), the Fundación Francisco Cobos, and the Asociación Española Contra el Cáncer (AECC).

The original article [1] has been corrected.
