# Supplementary figures and images for: iGlioSub: an integrative transcriptomic and epigenomic classifier for glioblastoma molecular subtypes
Source: BioData Min. 2021 Aug 23;14:42. doi: 10.1186/s13040-021-00273-8 (PMC8381510; doi:10.1186/s13040-021-00273-8)

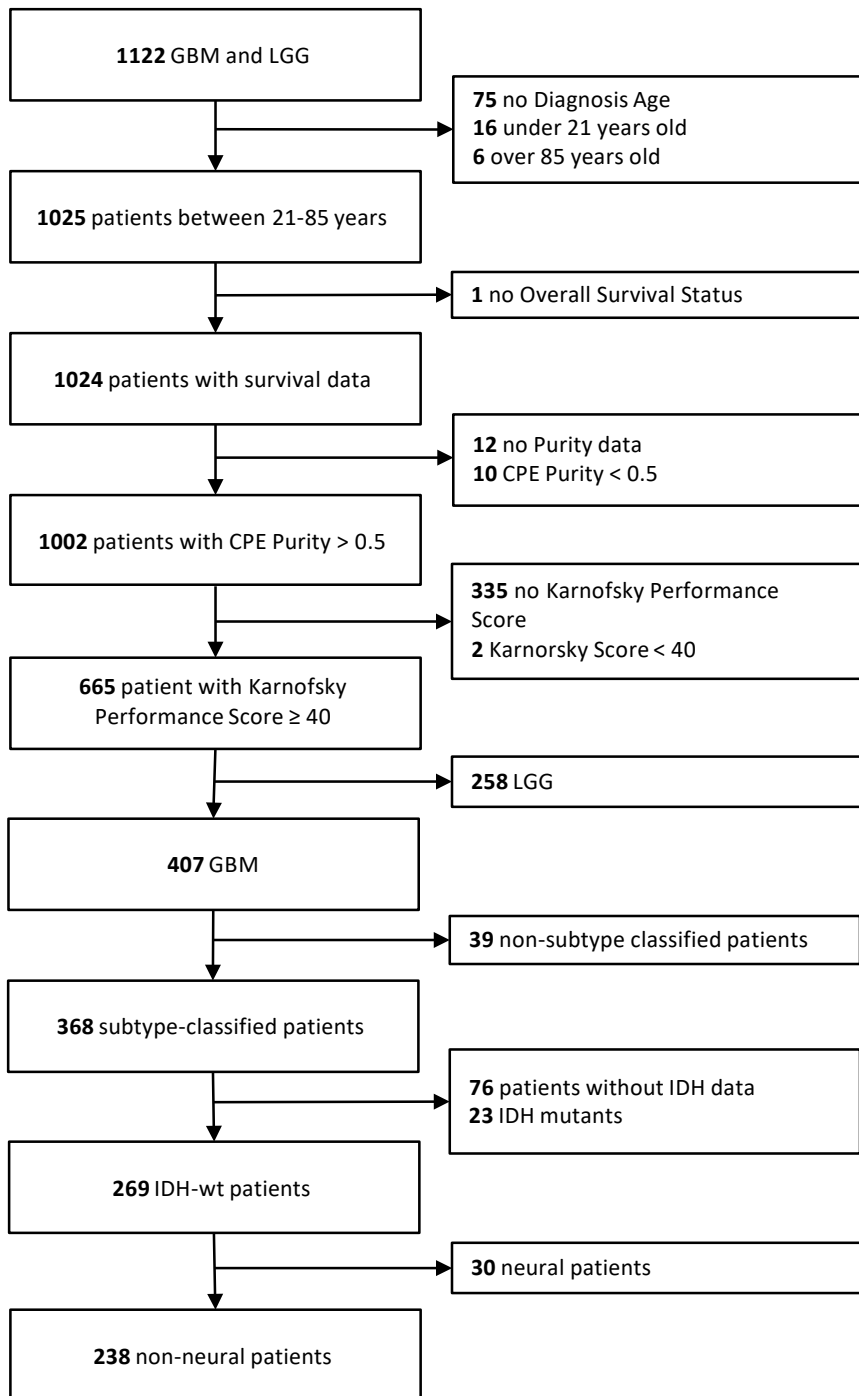

Supplement: Supplementary file 1 — Additional file 1: Suppl Figure 1. Process of curation of the TCGA-downloaded samples. [file 13040_2021_273_MOESM1_ESM.pdf]

A

All DEG

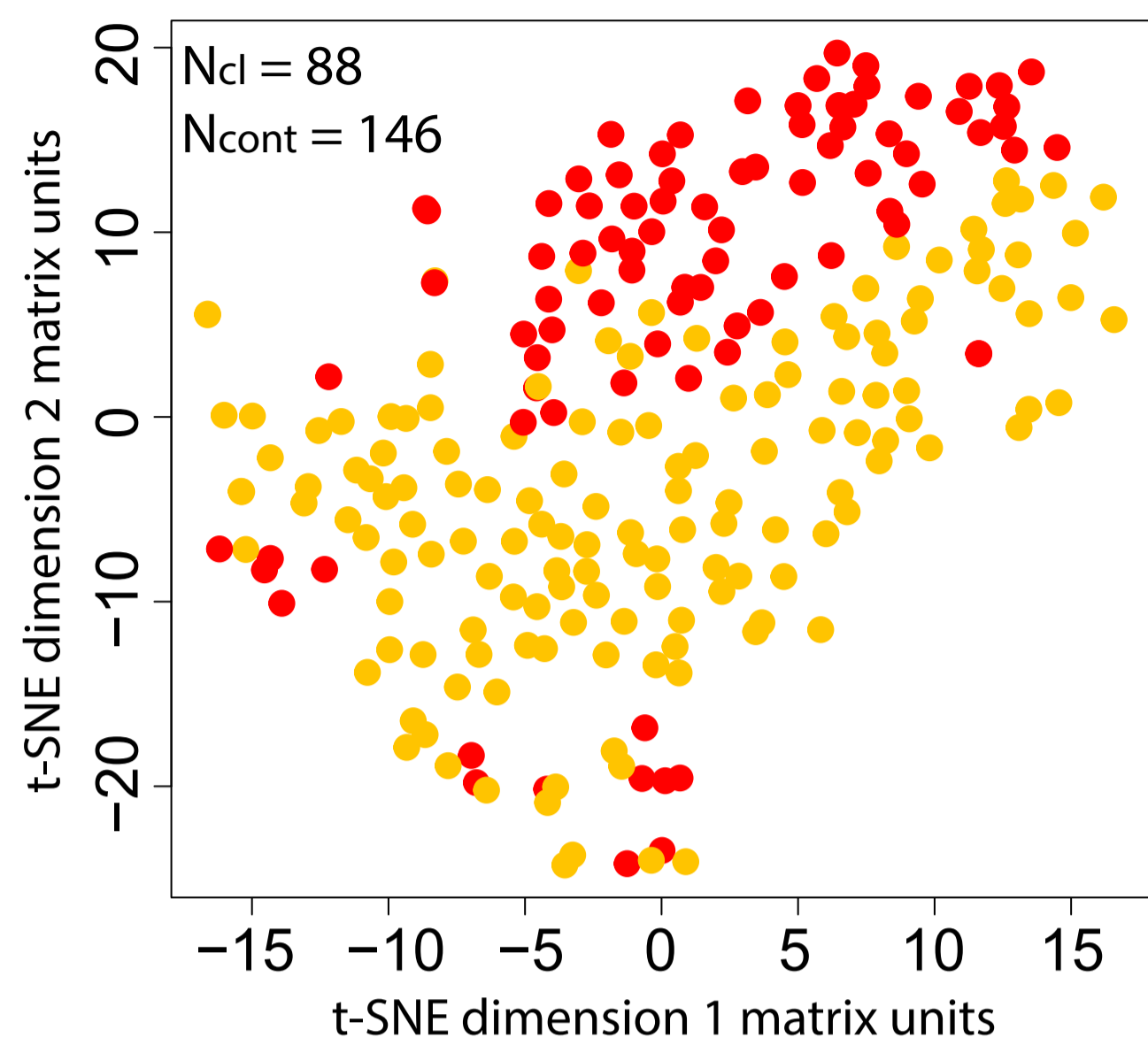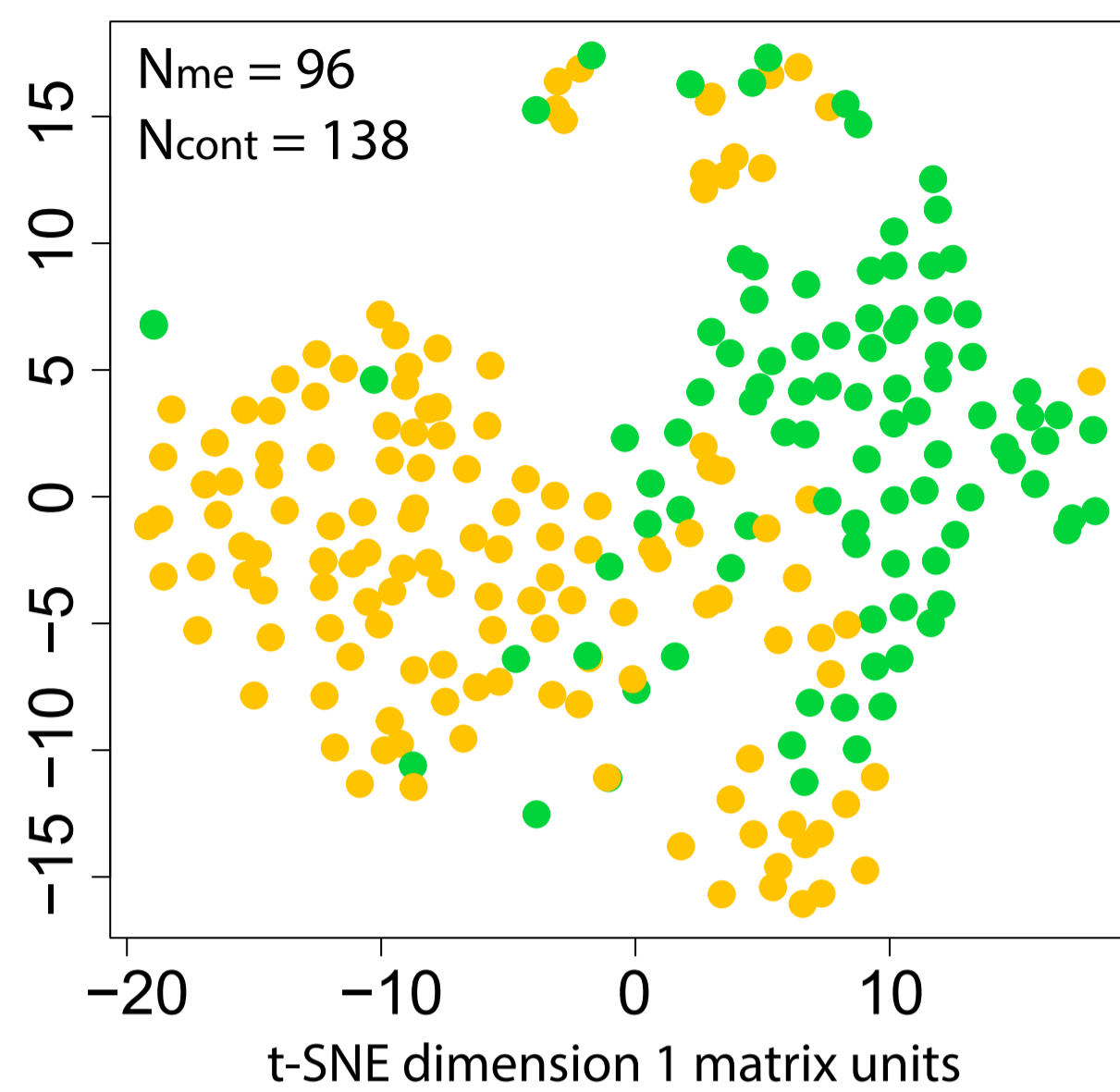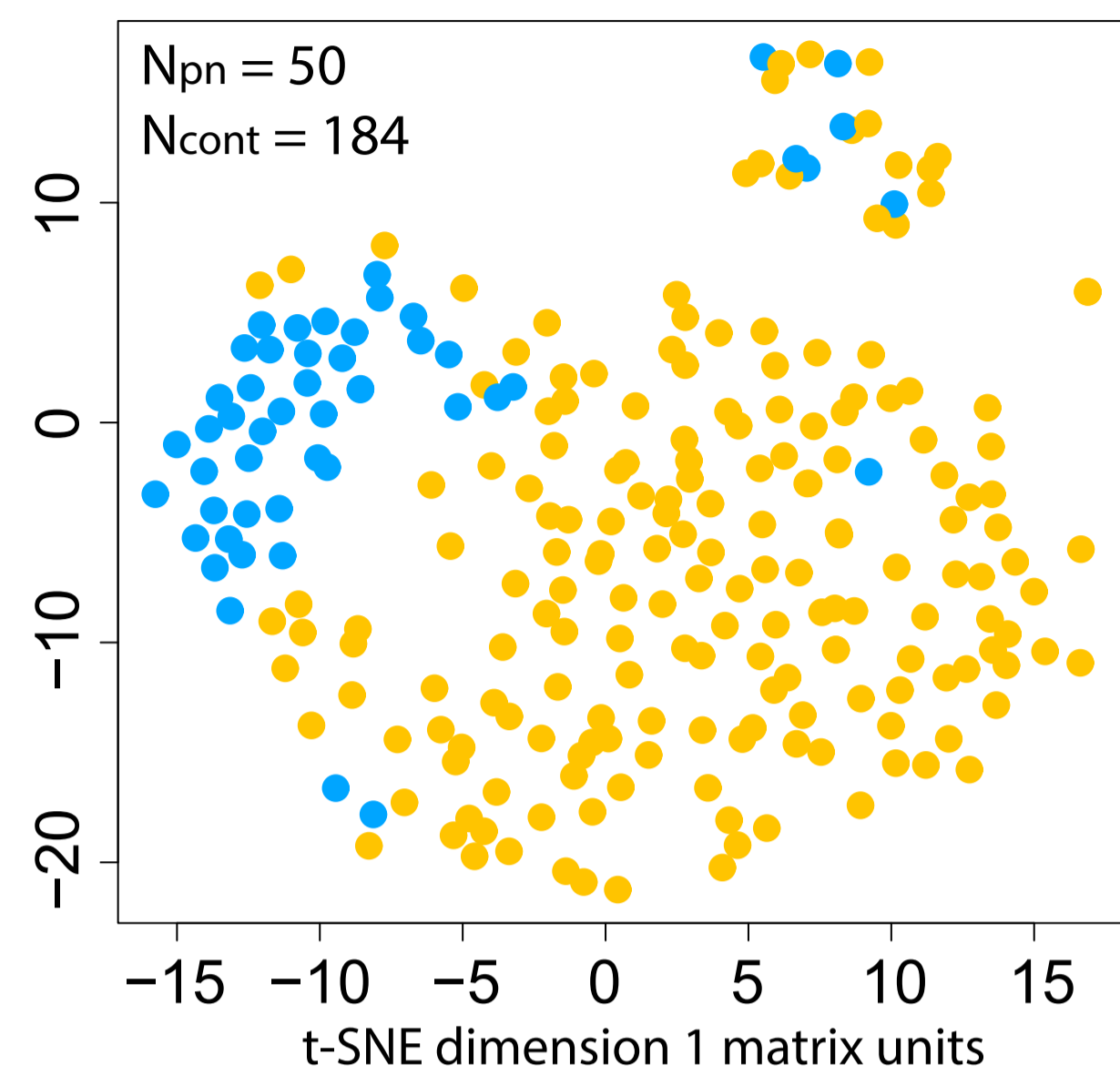

B

All DMS

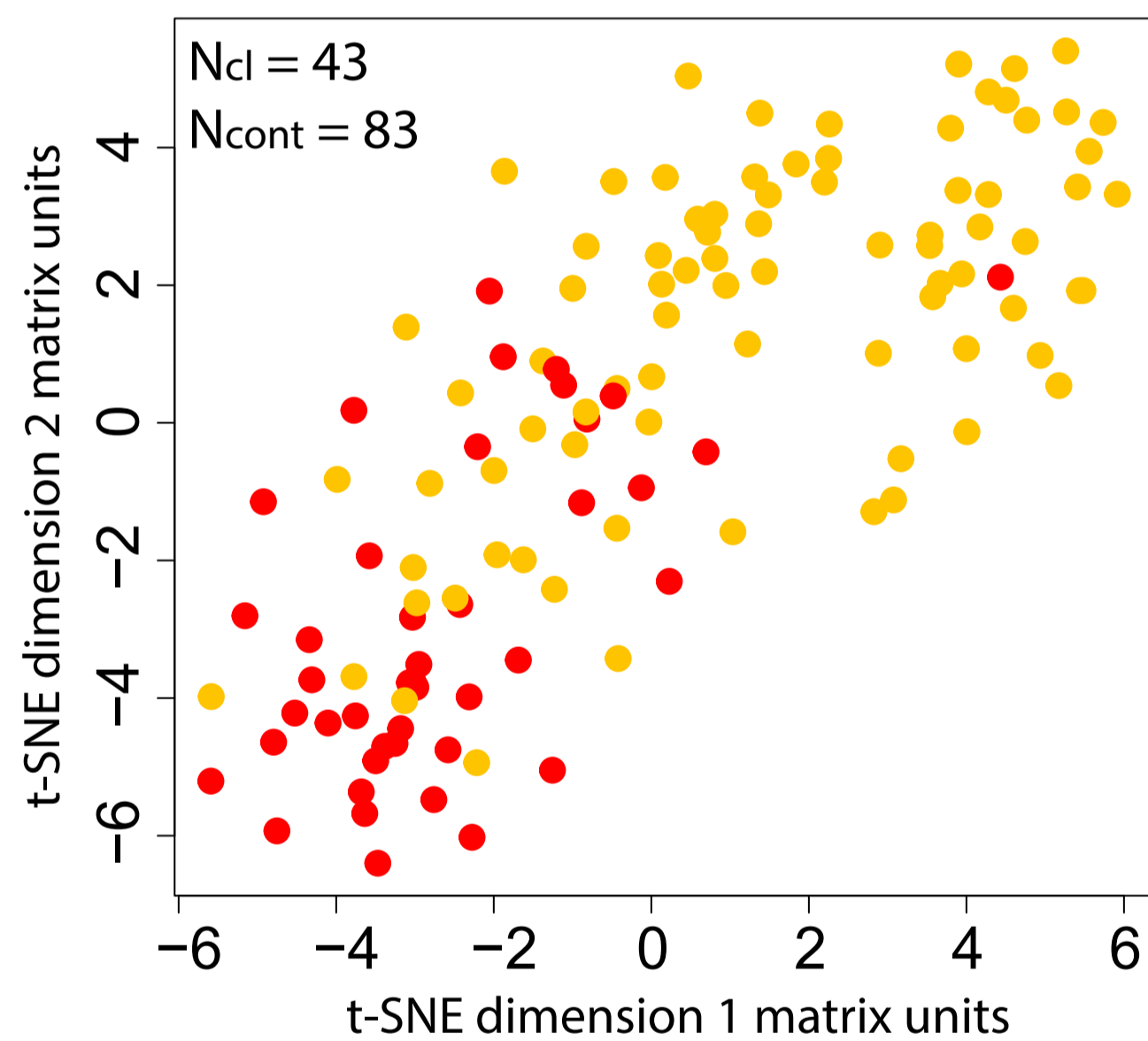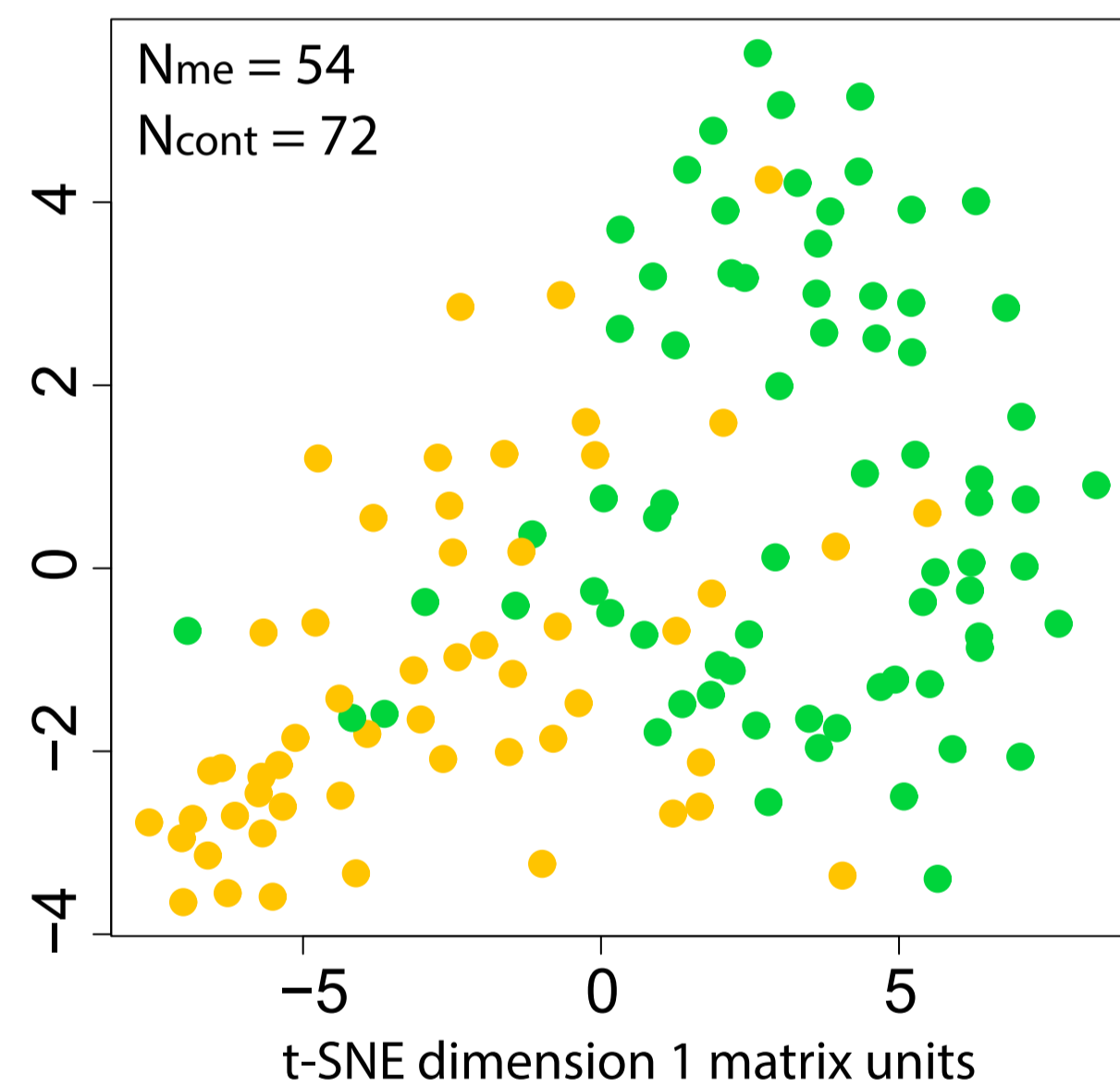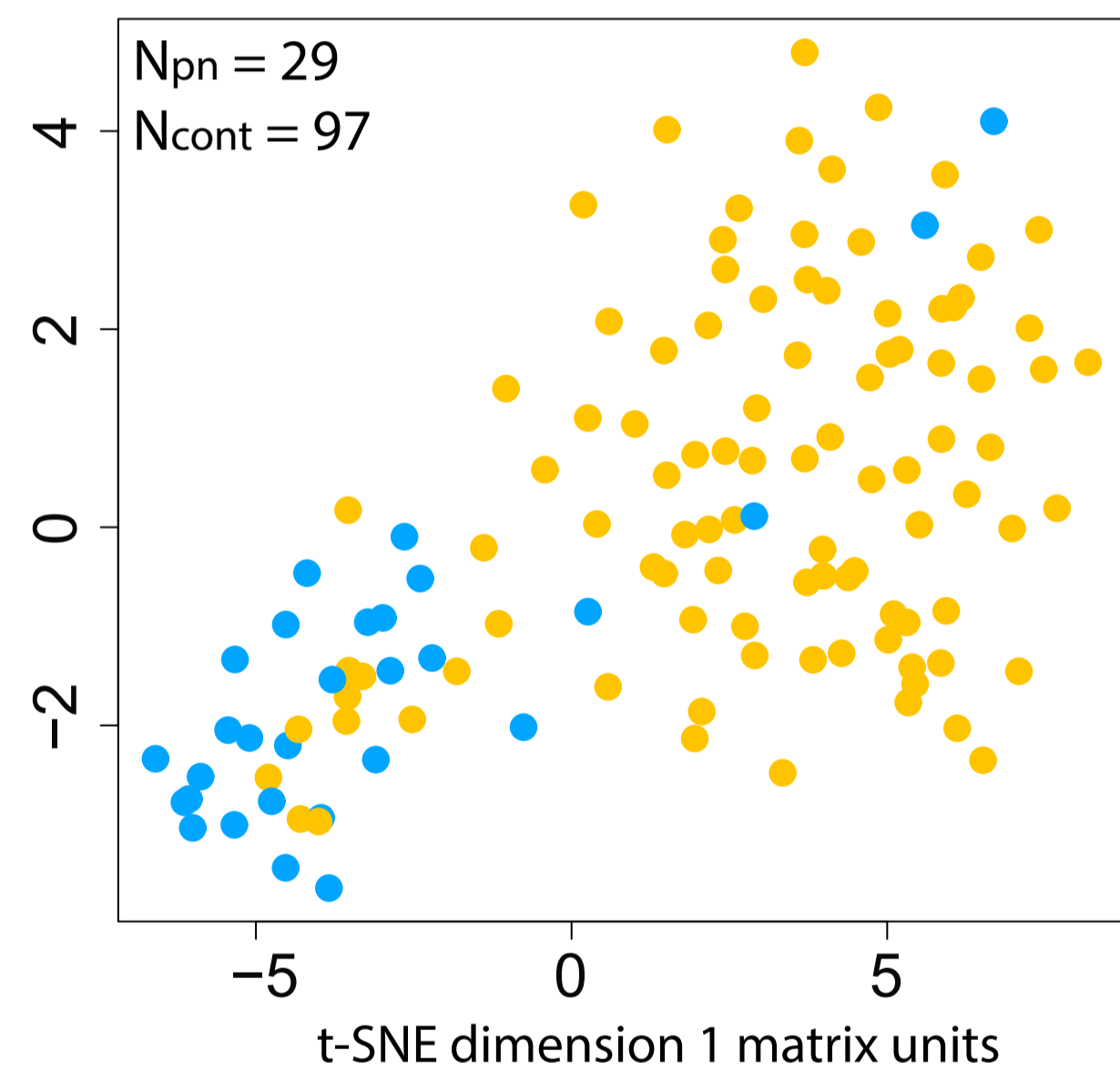

C

All DEG + All DMS

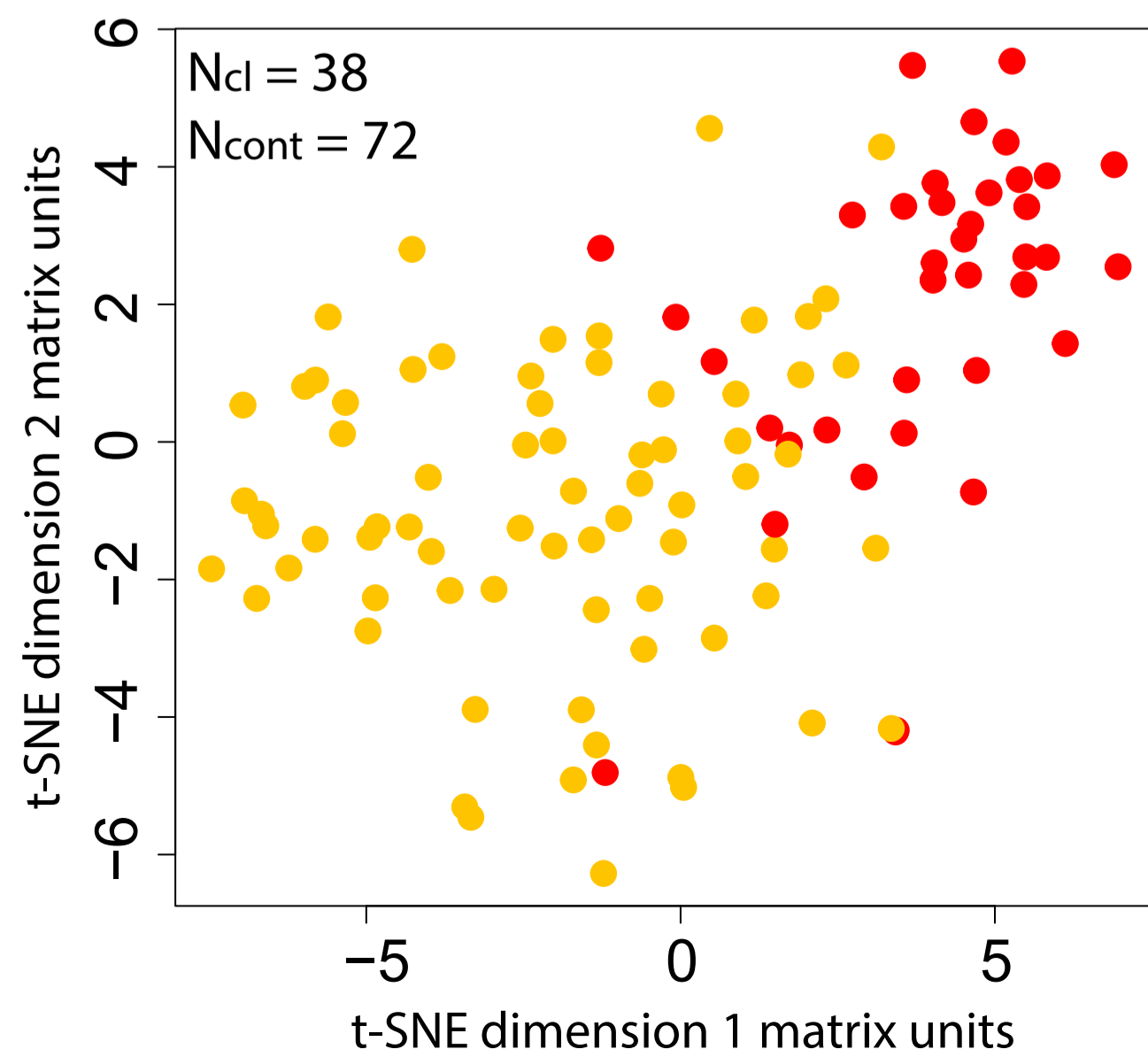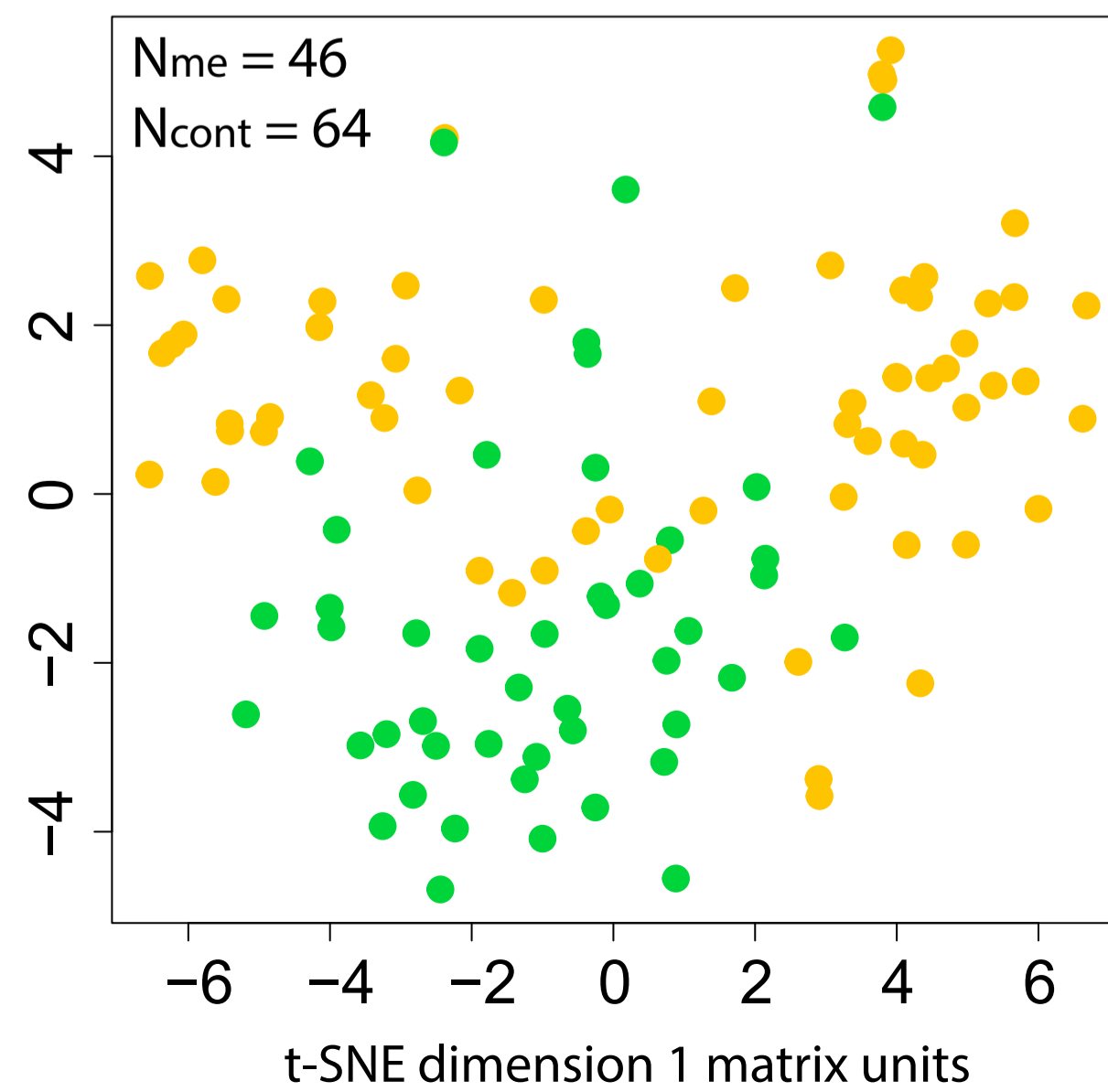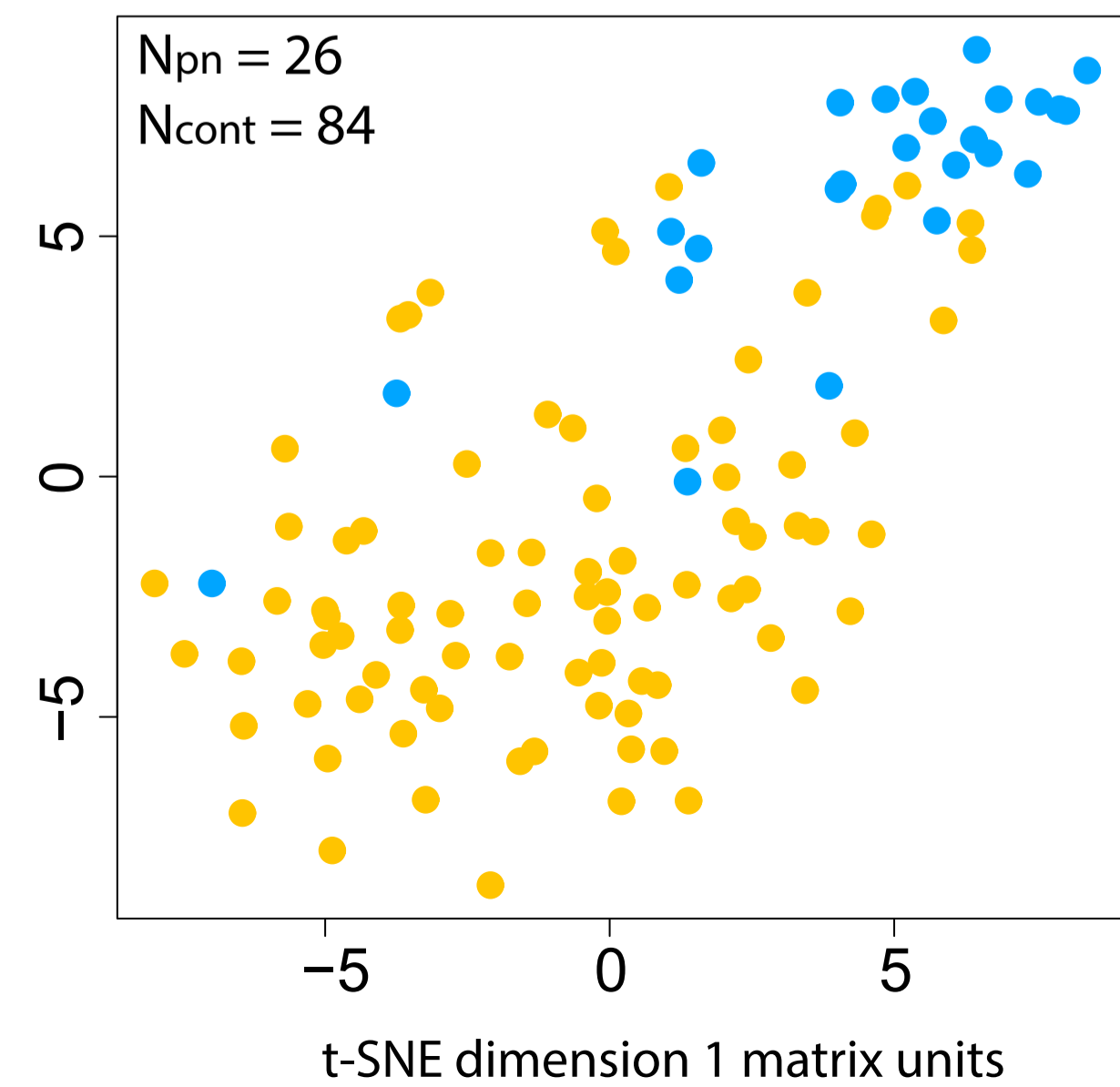

Supplement: Supplementary file 3 — Additional file 3: Suppl Figure 3. t-SNE plotting representing clustering of different subtype patients using (A) all differentially expressed genes (DEG), (B) all differentially methylated sites (DMS), or (C) all DEG and DMS. All plots show a modest classification potential using gene expression data of all DEG, DNAm data of all DMS, or both all DEG and DMS combined. [file 13040_2021_273_MOESM3_ESM.pdf]

A

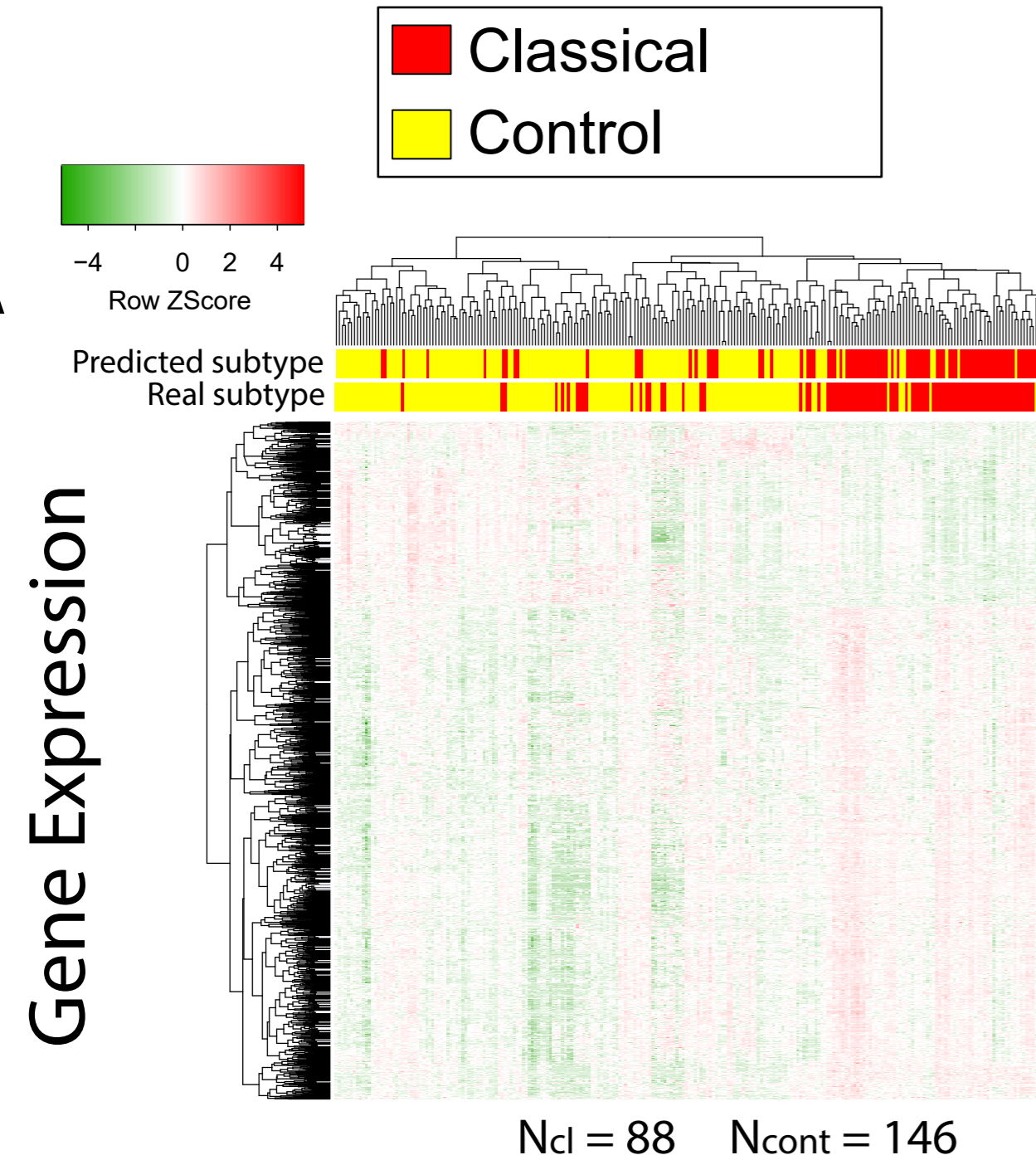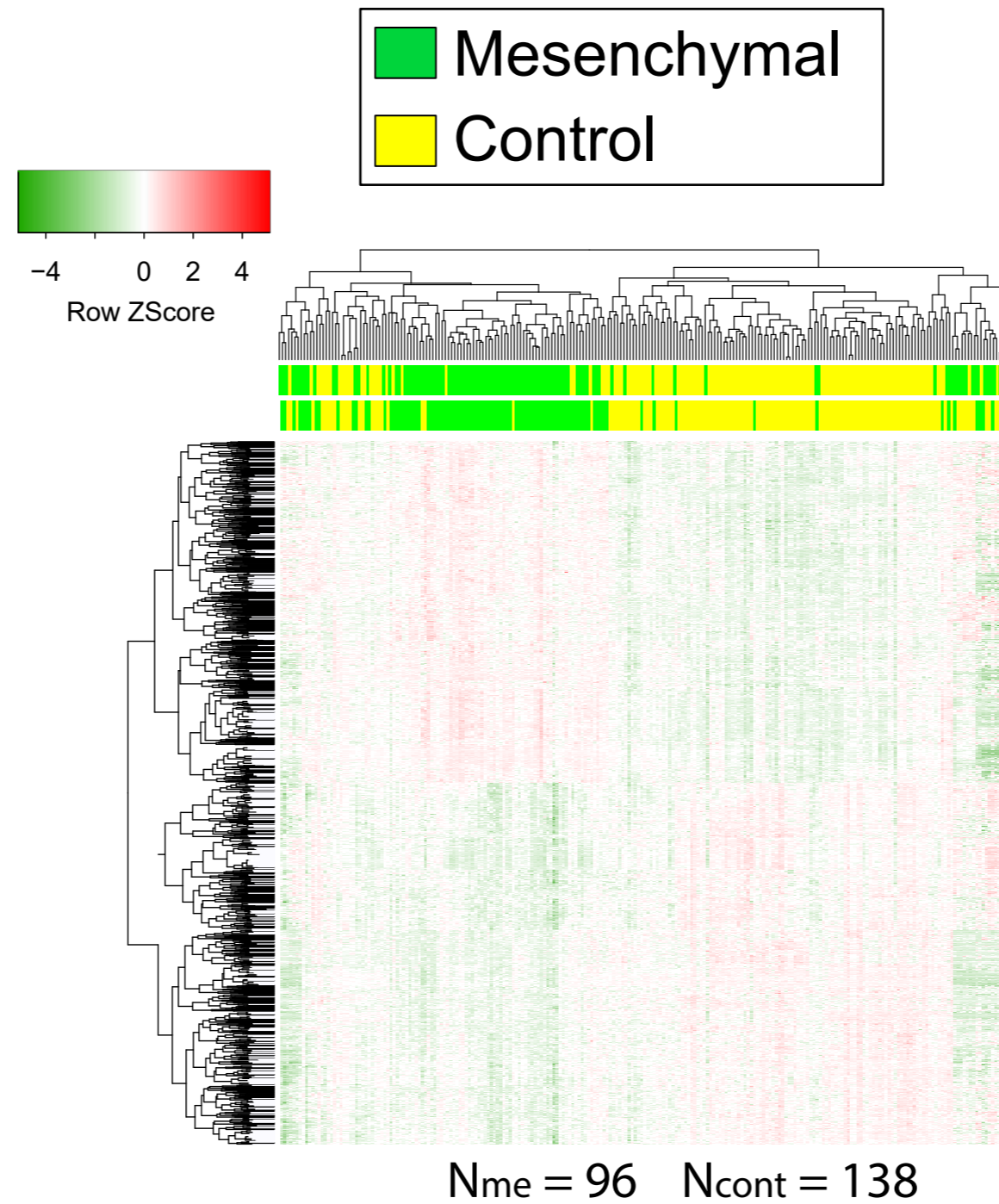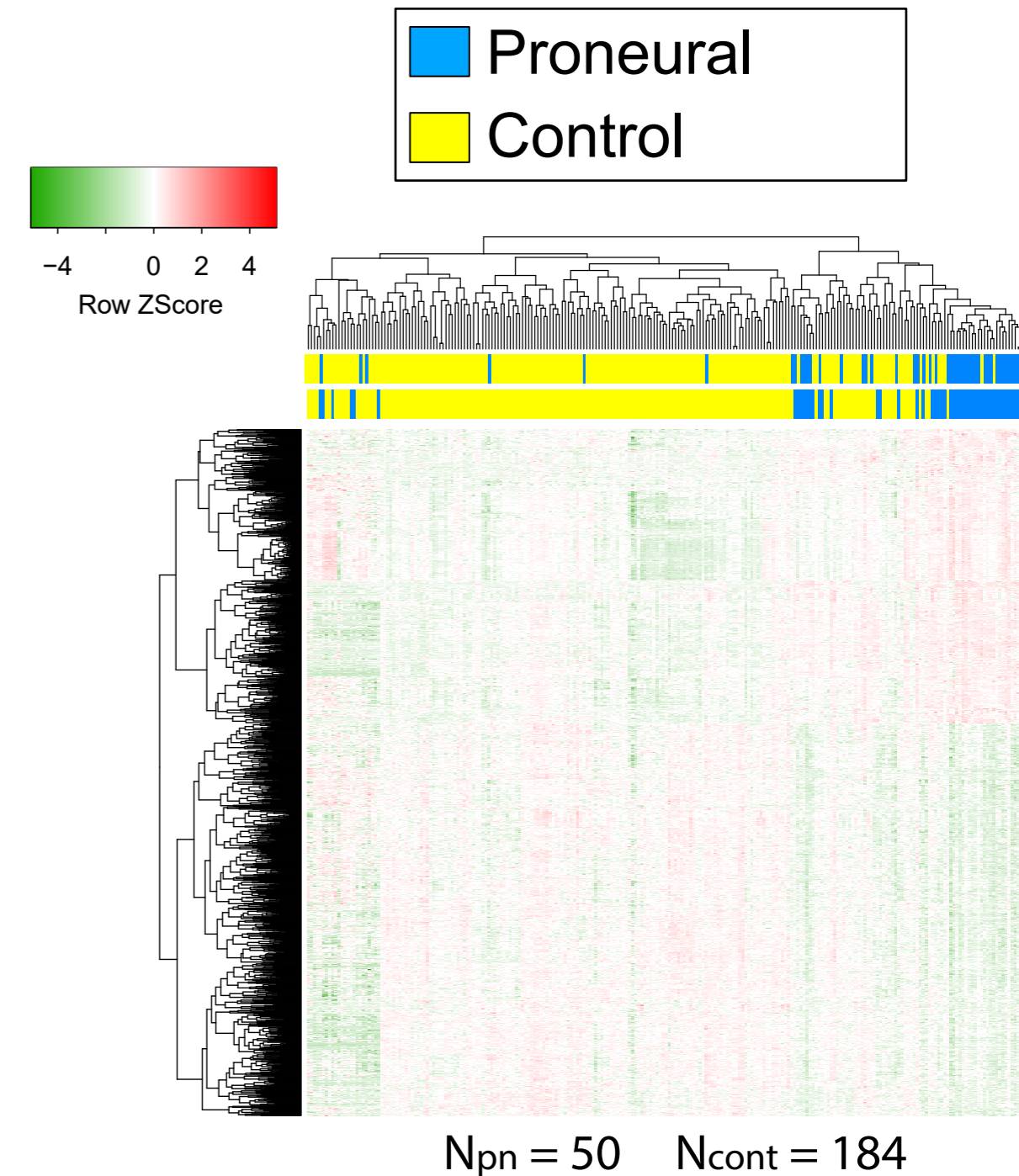

B

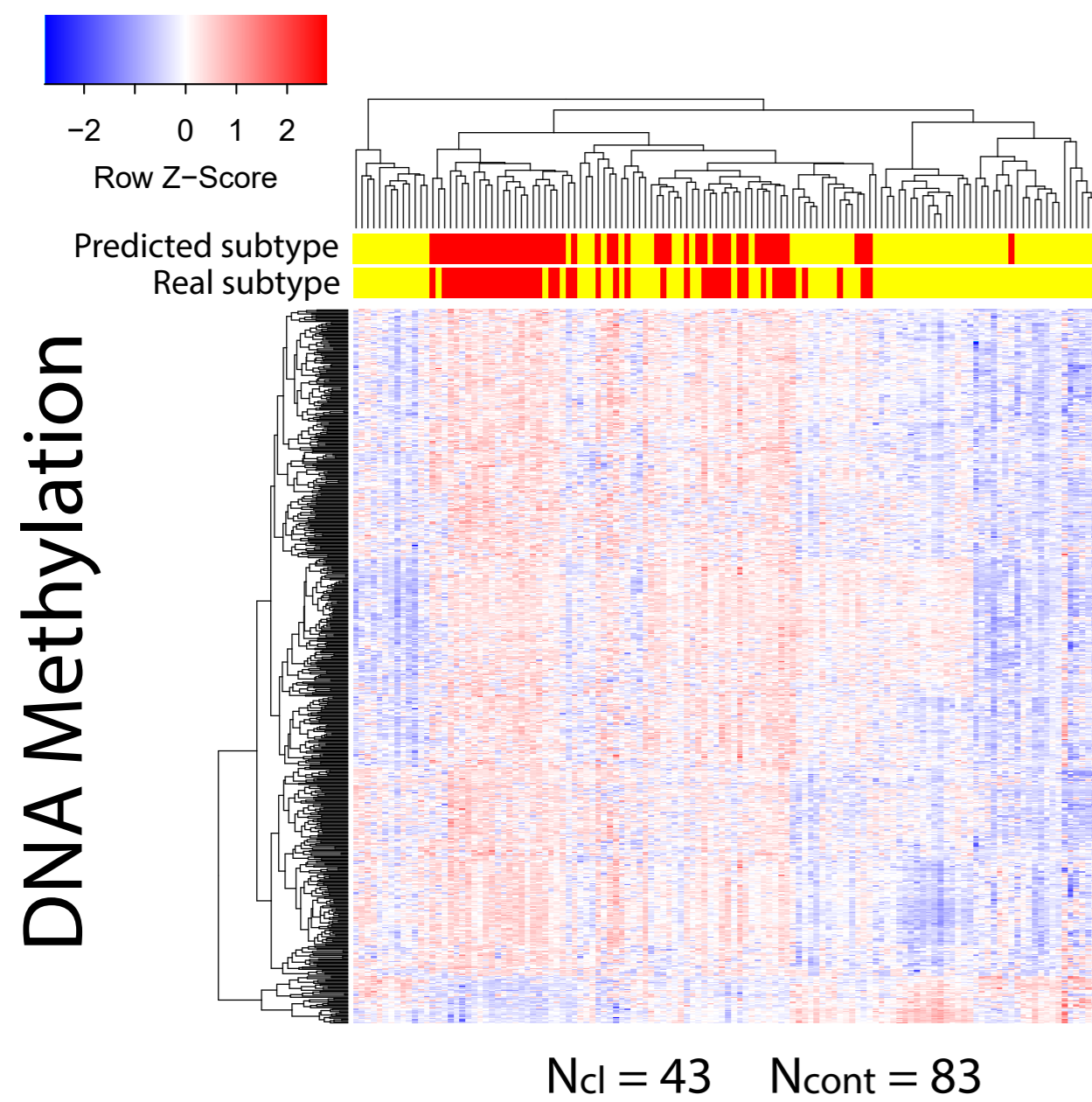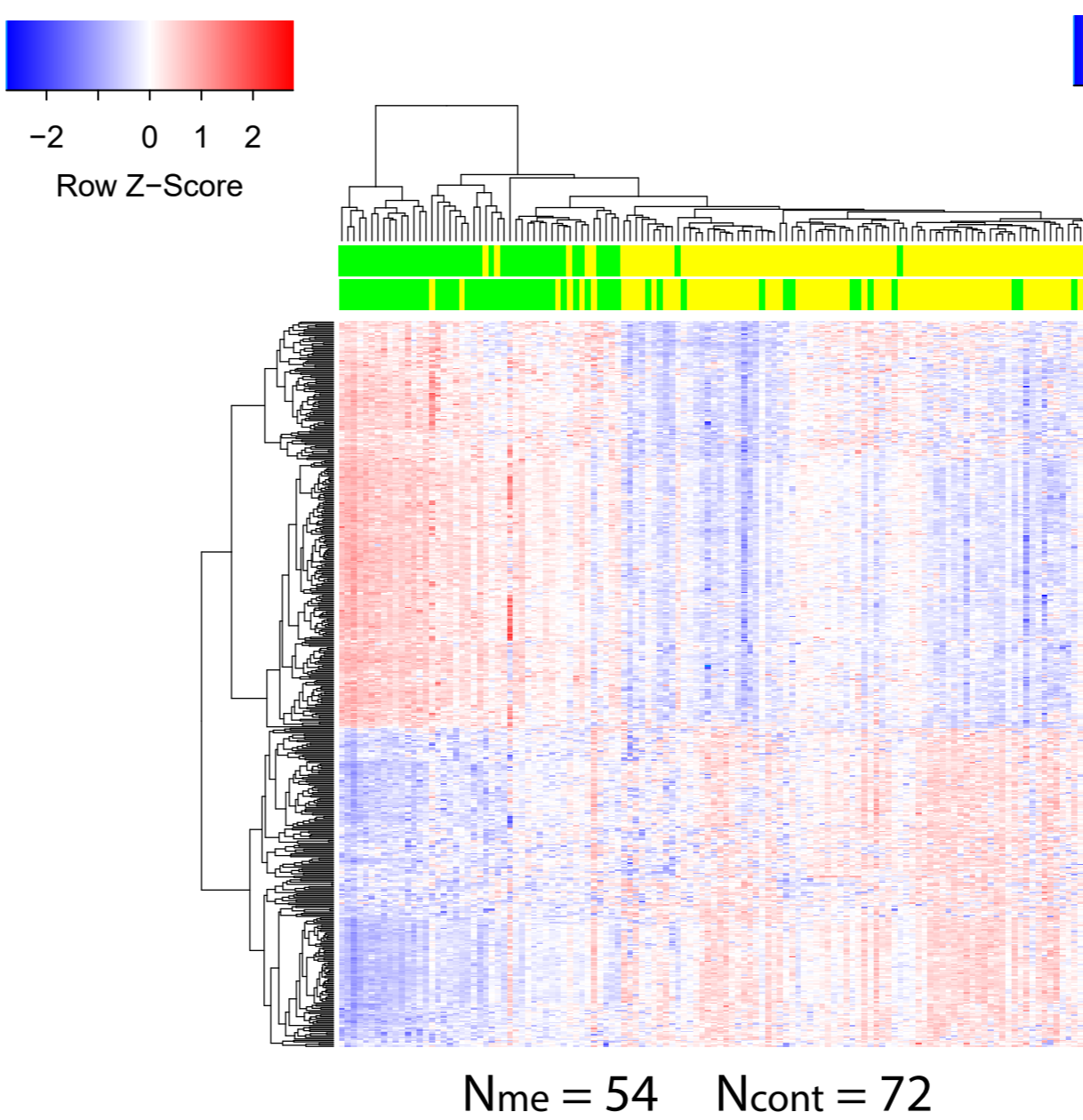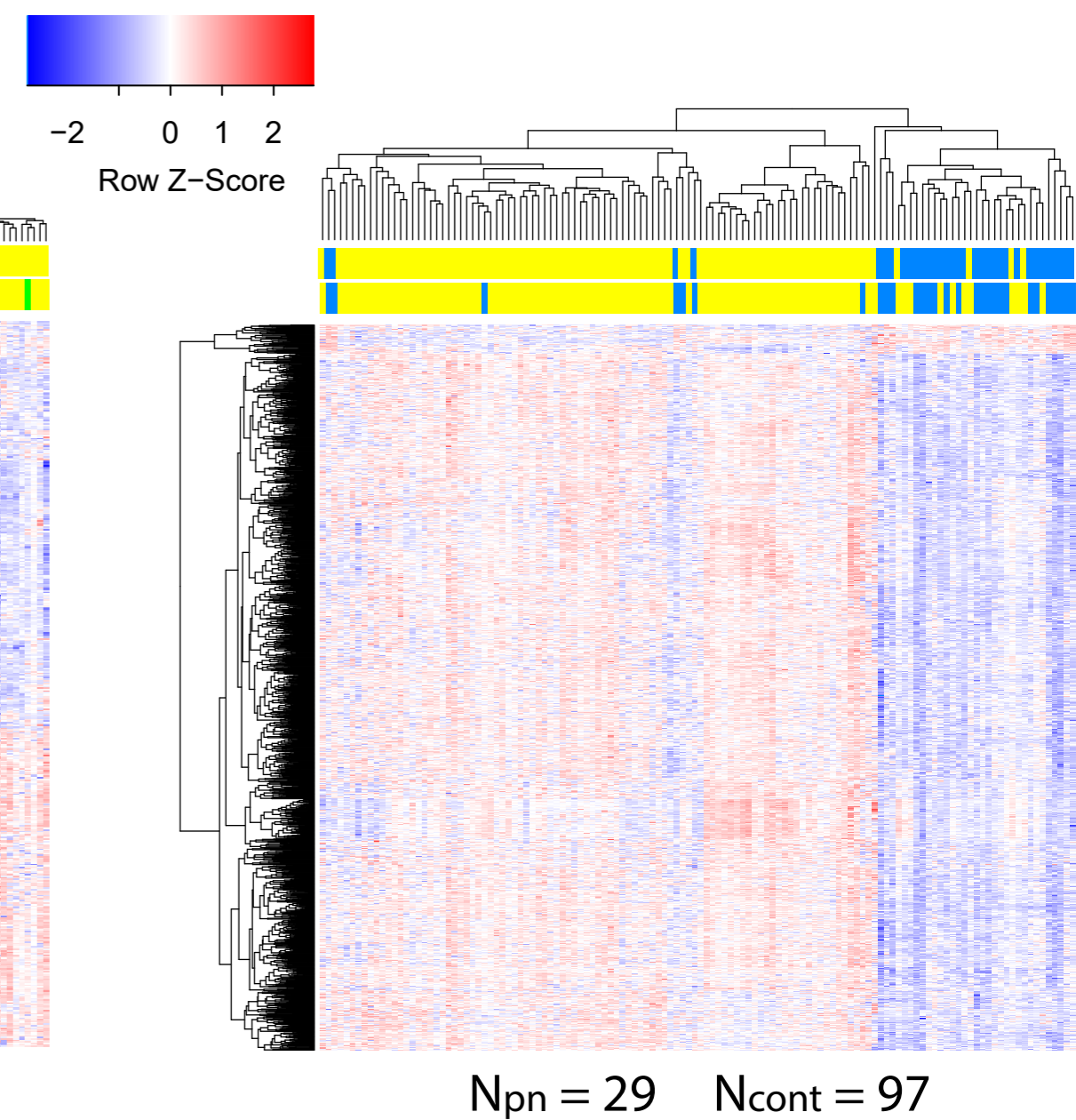

Supplement: Supplementary file 4 — Additional file 4: Suppl Figure 4. Hierarchical cluster analysis using Euclidean distance for the gene expression/DNAm levels of (A) all differentially expressed genes (DEG), (B) all differentially methylated sites (DMS), or (C) all DEG and DMS. All plots show a significant classification potential of hierarchical clustering using gene expression data of all DEG, DNAm data of all DMS, or both all DEG and DMS combined. [file 13040_2021_273_MOESM4_ESM.pdf]

Gene Expression

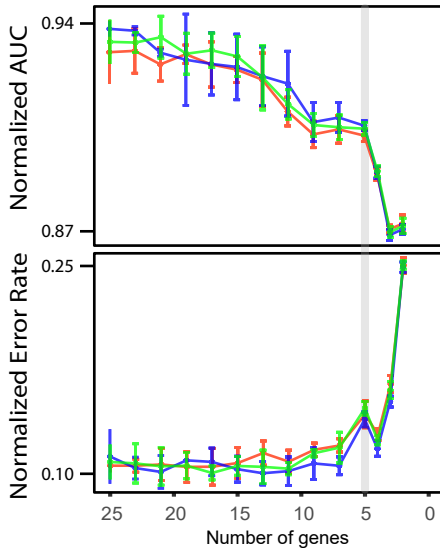

DNAm

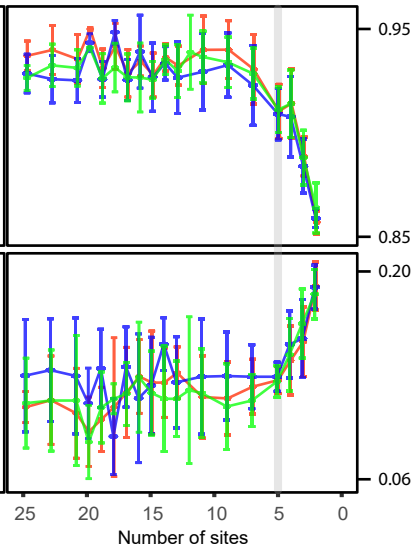

Classical

Mesenchymal

Proneural

Supplement: Supplementary file 5 — Additional file 5: Suppl Figure 5. Comparison of the diminution of the Area Under Curve (AUC) (top panels) and increase of the Error Rate (bottom panels) with less than five features per panel for the gene expression (left) and DNAm (right) panels. The lines represent the mean AUC±SD and Error Rate±SD for each subtype. All lines are normalized against the range of the classifier with the highest AUC or the lowest error rate (Classical subtype classifier). Both AUC and Error Rate show an optimal performance when using five features per panel. [file 13040_2021_273_MOESM5_ESM.pdf]

# Gene Expression

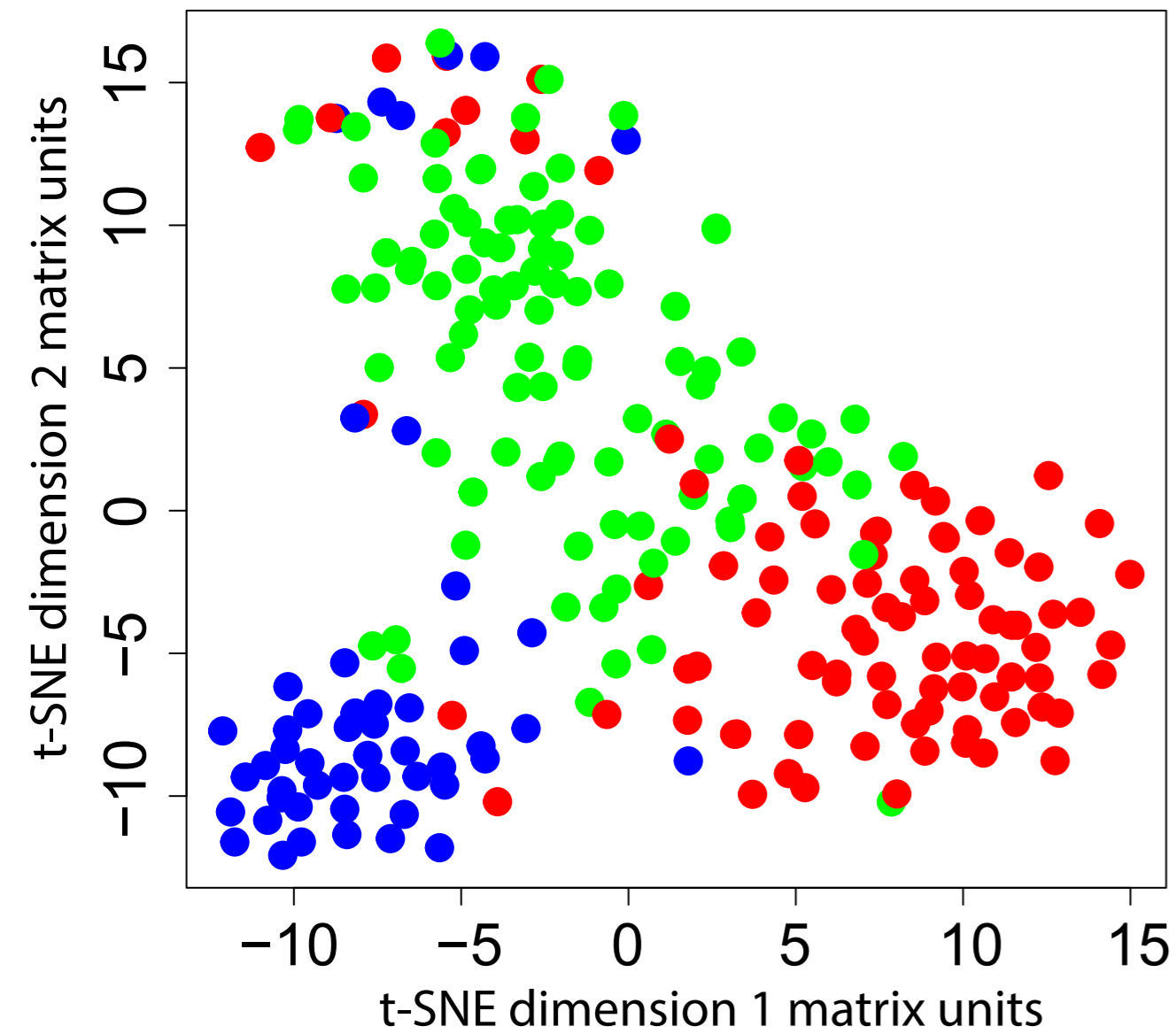

# DNA methylation

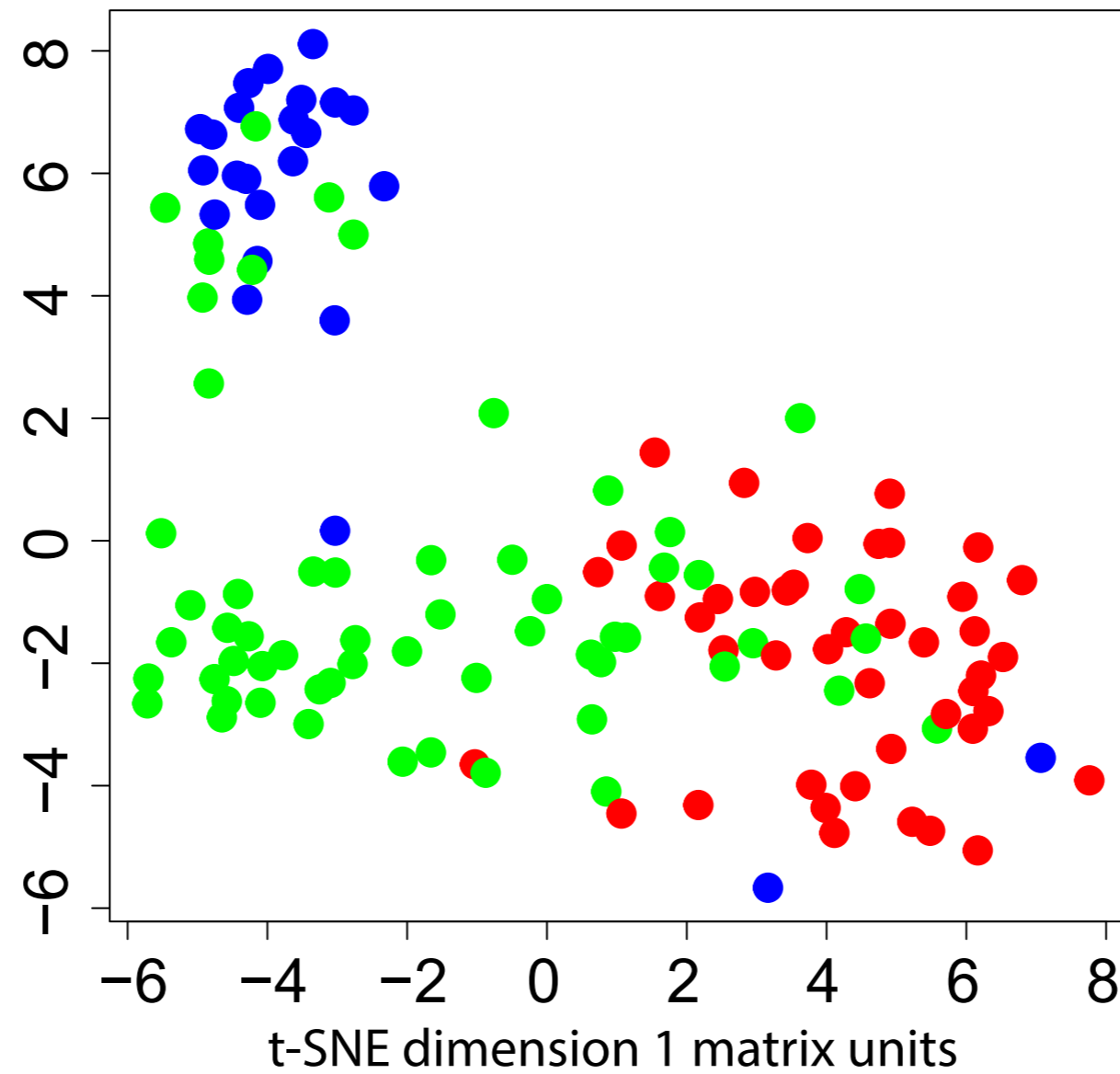

# iGlioSub

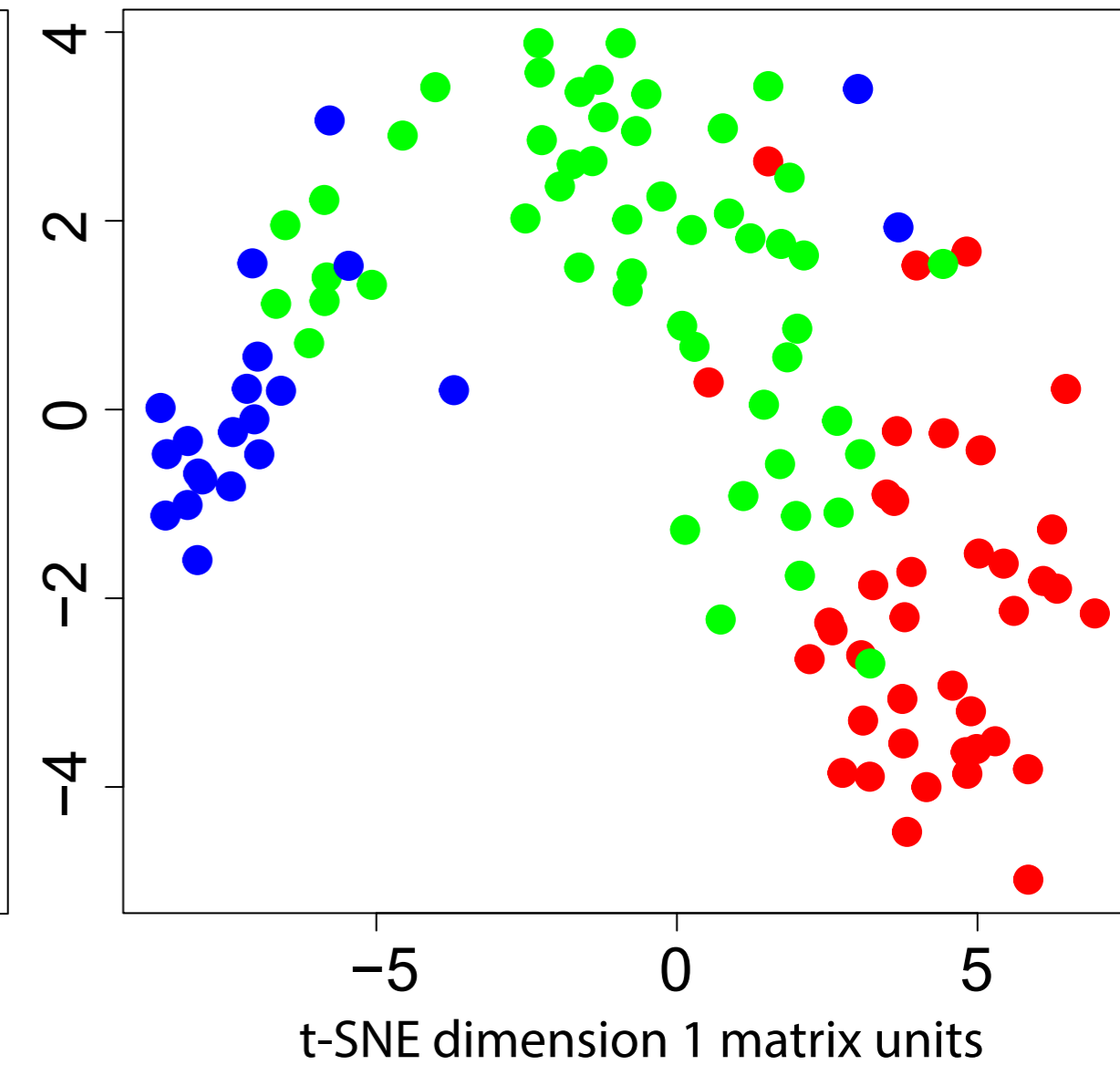

Subtype

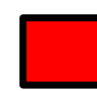

Classical

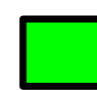

Mesenchymal

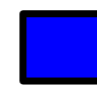

Proneural

Supplement: Supplementary file 6 — Additional file 6: Suppl Figure 6. t-SNE plots representing clustering of different subtypes using the combination of the three-subtype panels using gene expression, DNAm, and iGlioSub. [file 13040_2021_273_MOESM6_ESM.pdf]

# Gene Expression

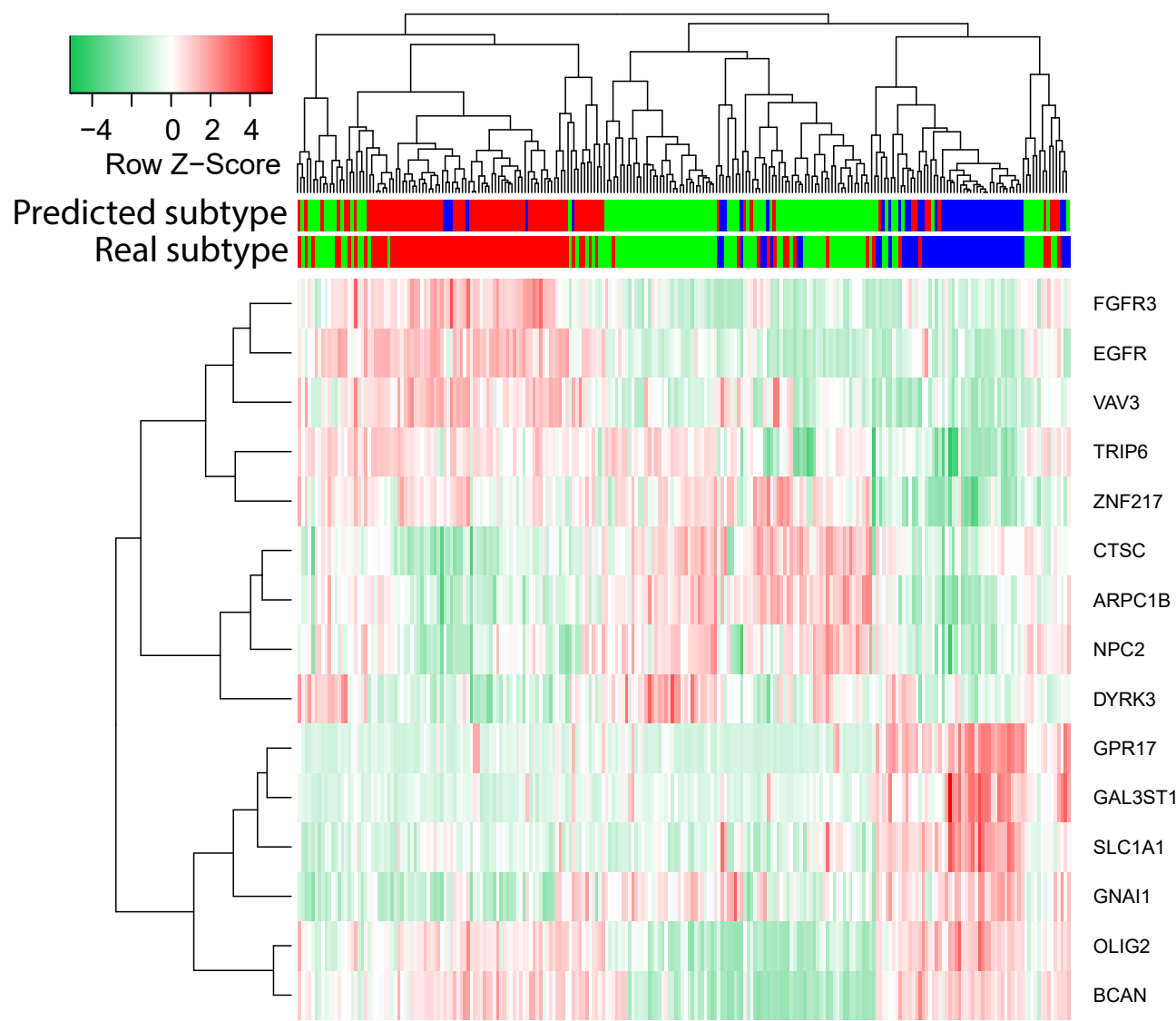

# DNA methylation

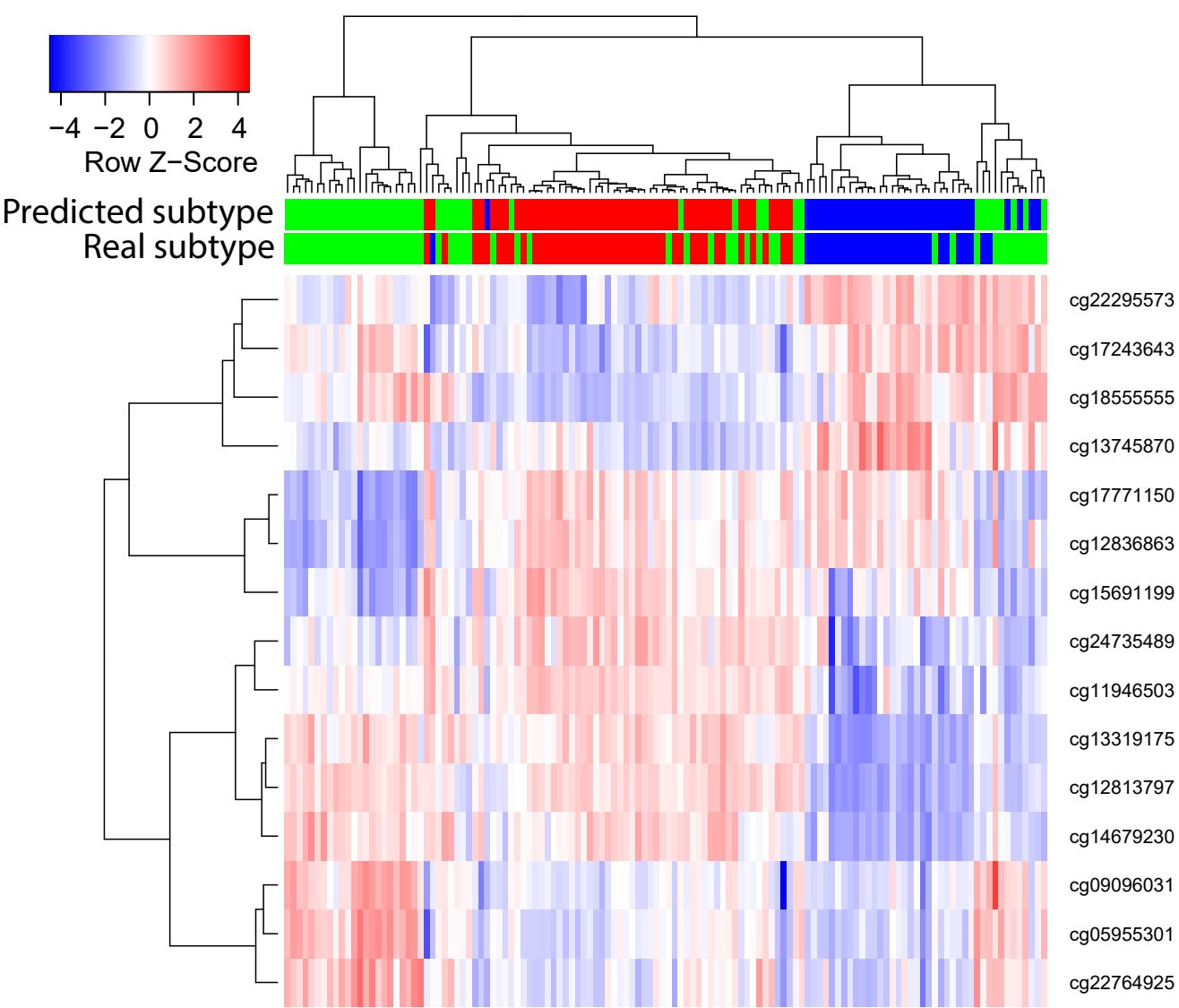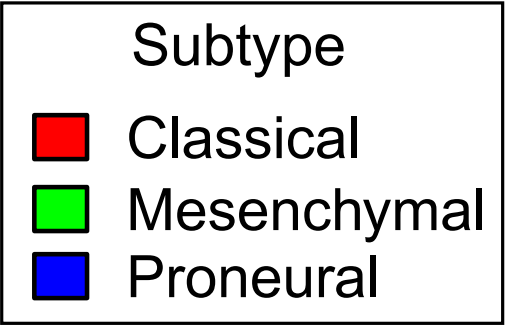

Supplement: Supplementary file 7 — Additional file 7: Suppl Figure 7. Hierarchical cluster analysis using Euclidean distance for the gene expression/DNAm levels of the three-subtype panels simultaneously (n = 15 features). [file 13040_2021_273_MOESM7_ESM.pdf]

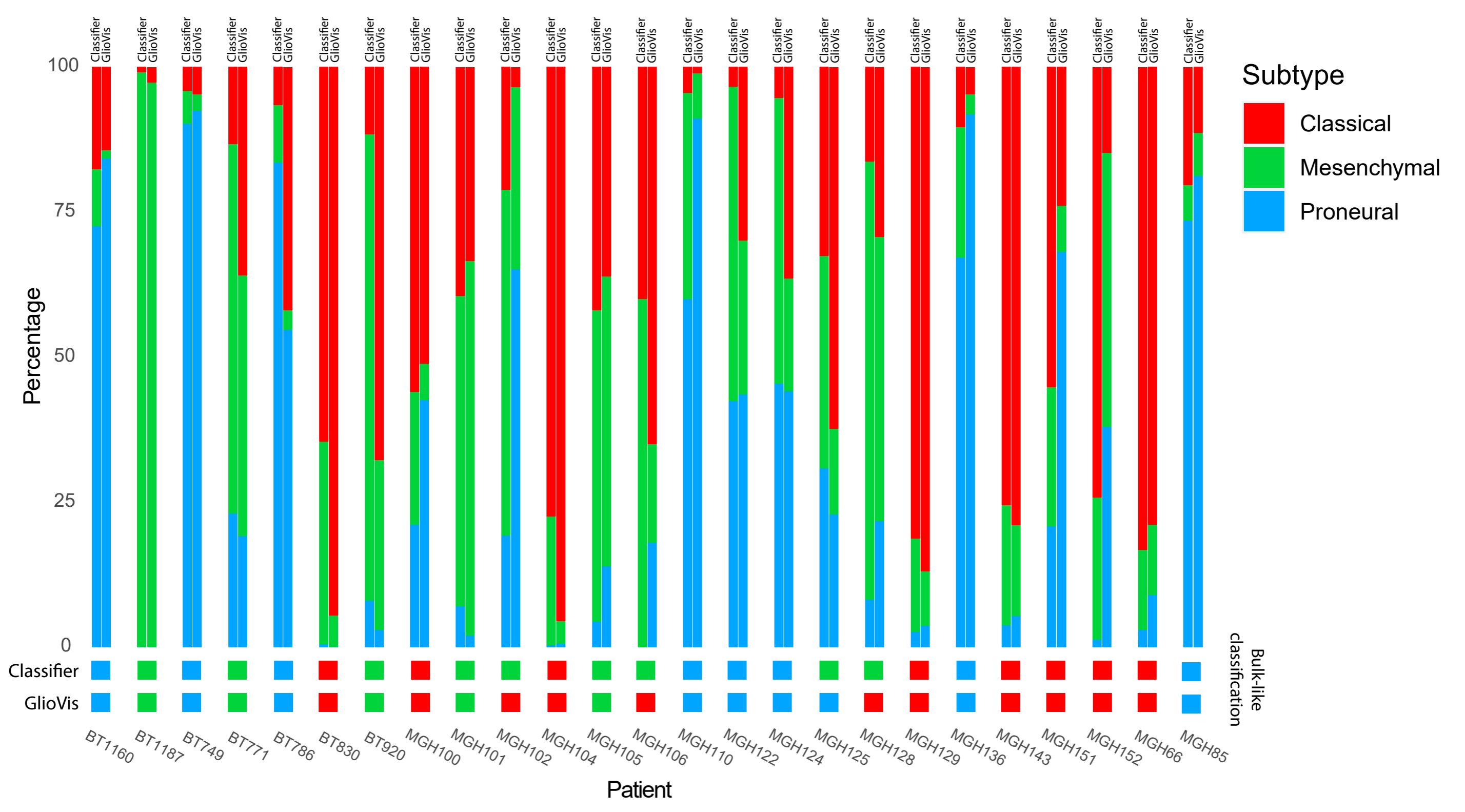

Supplement: Supplementary file 8 — Additional file 8: Suppl Figure 8. Barplots displaying the percentage of cells classified as Classical, Mesenchymal, and Proneural from a single-cell RNA-seq experiment using our gene expression-based classifier and GlioVis. The colored squares represent the subtype selected by the classifier and GlioVis for each patient using a bulk simulation using the mean expression of all cells. Both methodologies display a moderate agreement in the selection of the predominant subtype (κ=0.45), and a significant agreement between the predominant subtype and the assigned subtype using the bulk-like method (classifier κ=0.88; GlioVis κ=0.63). [file 13040_2021_273_MOESM8_ESM.pdf]

Classical

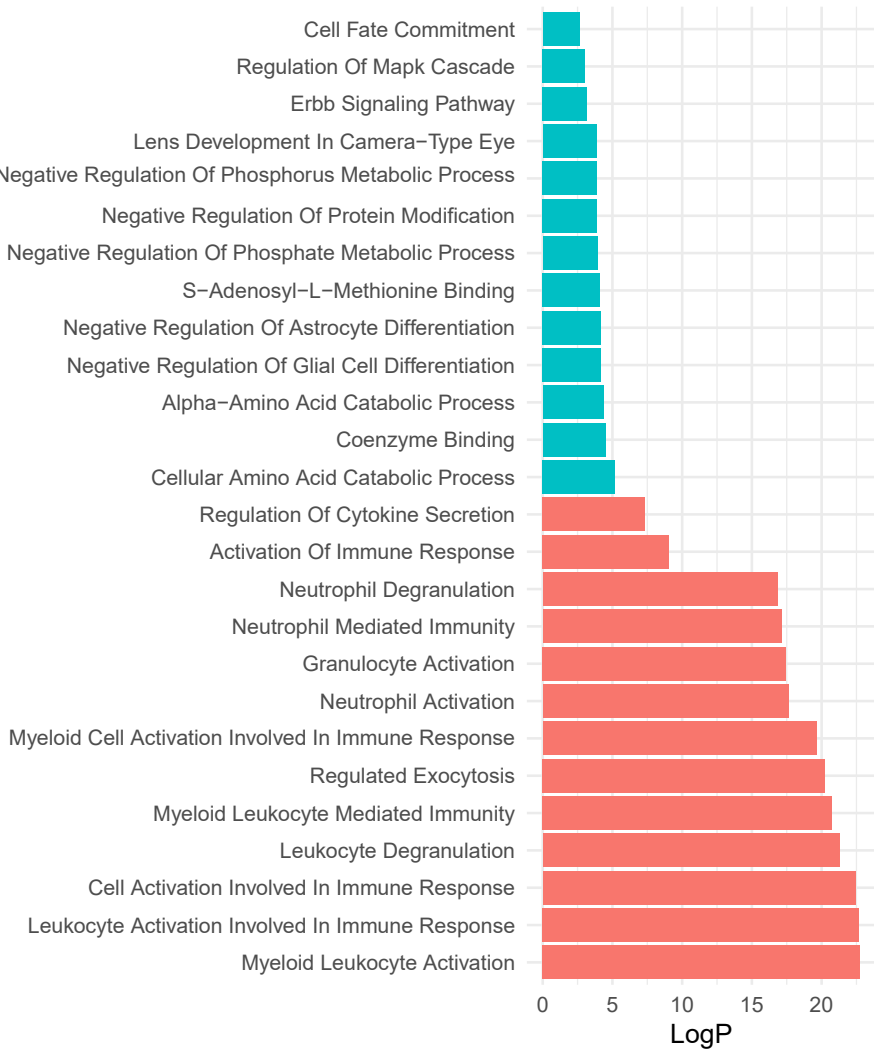

Mesenchymal

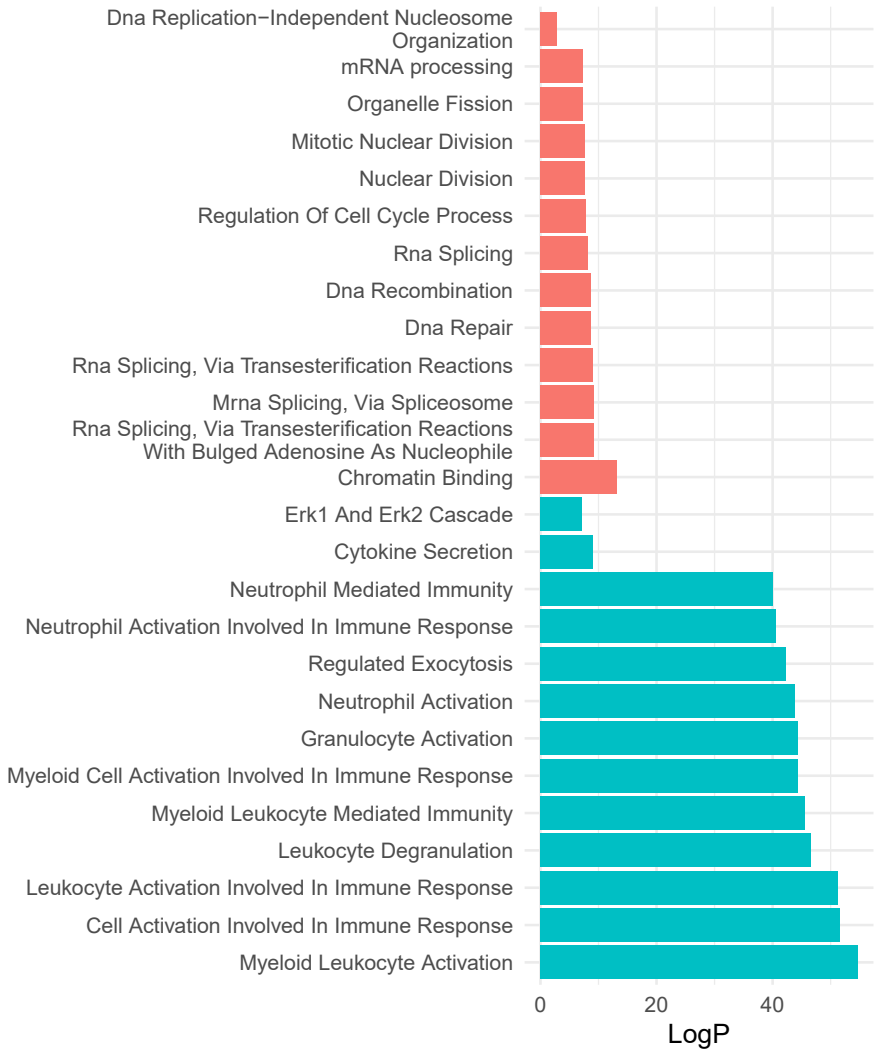

Proneural

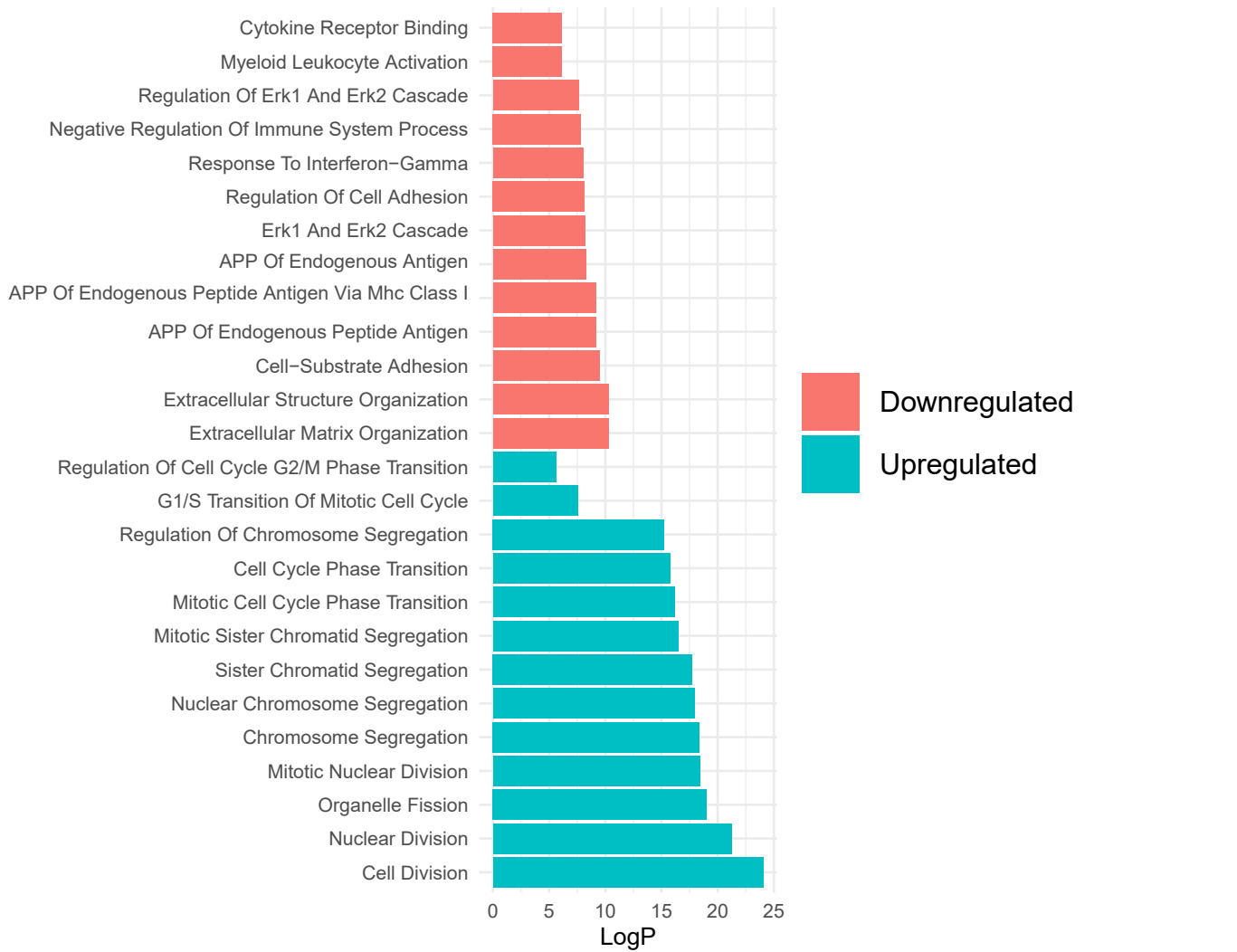

Downregulated

Upregulated

Supplement: Supplementary file 9 — Additional file 9: Suppl Figure 9. Pathways enriched for upregulated and downregulated genes in Classical, Mesenchymal, and Proneural subtype patients compared to the rest of patients. [file 13040_2021_273_MOESM9_ESM.pdf]

# Classical

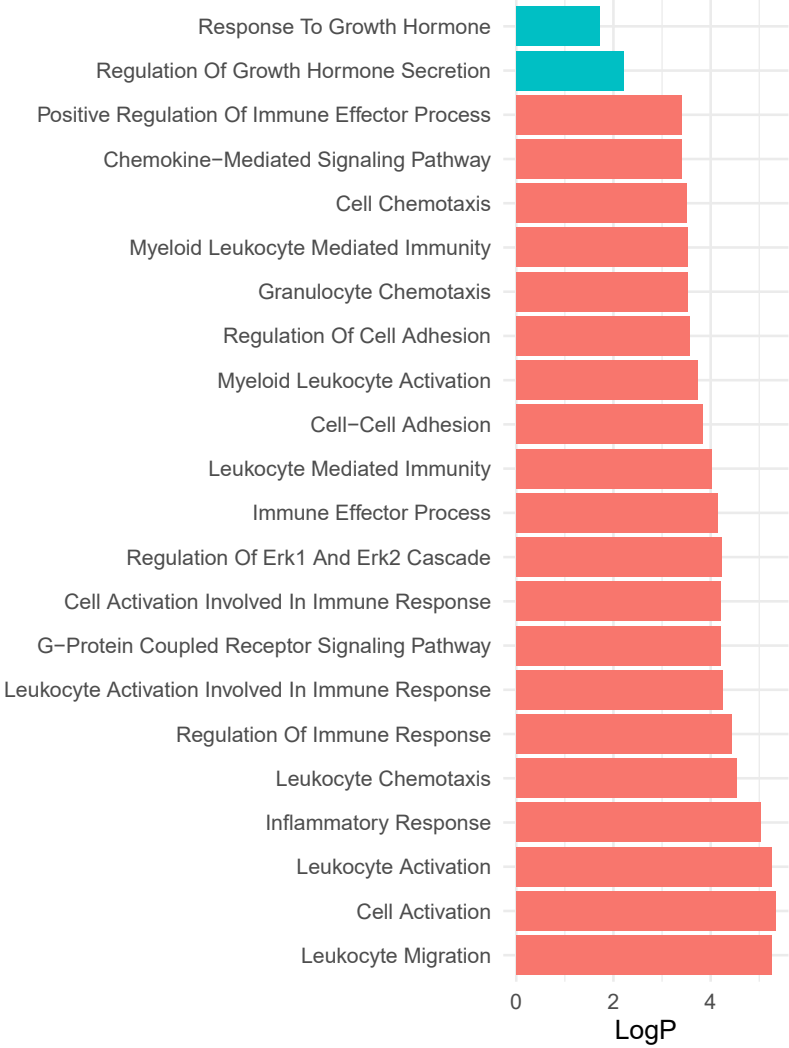

# Mesenchymal

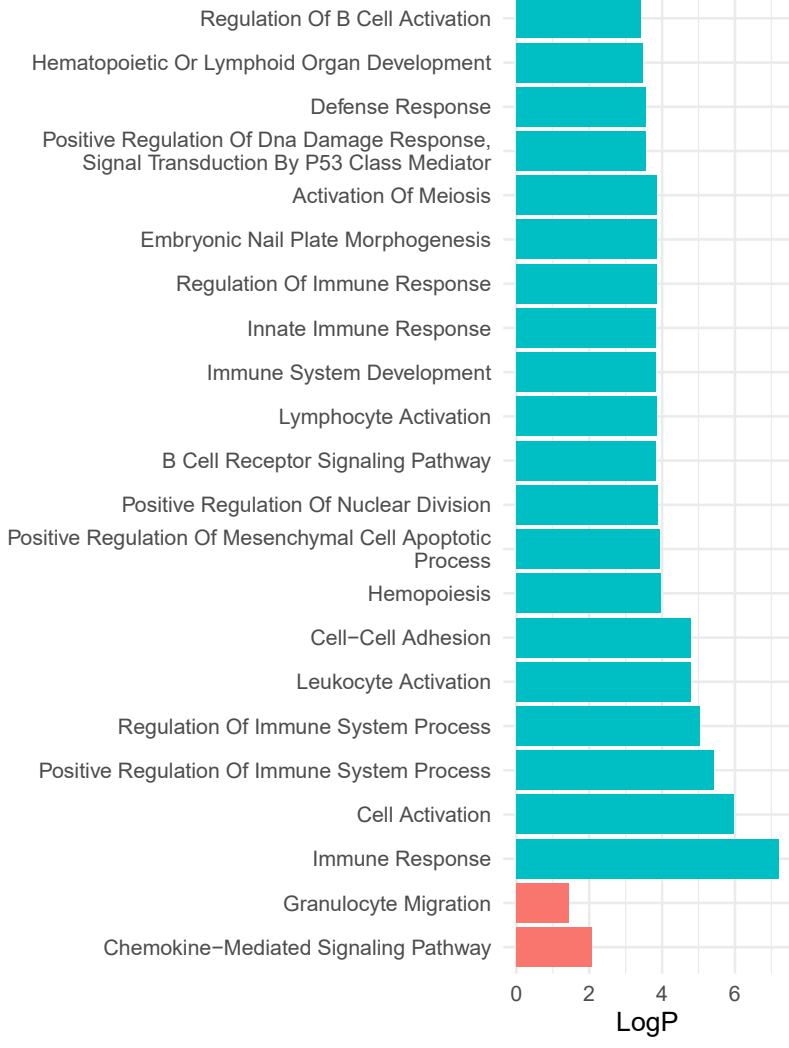

# Proneural

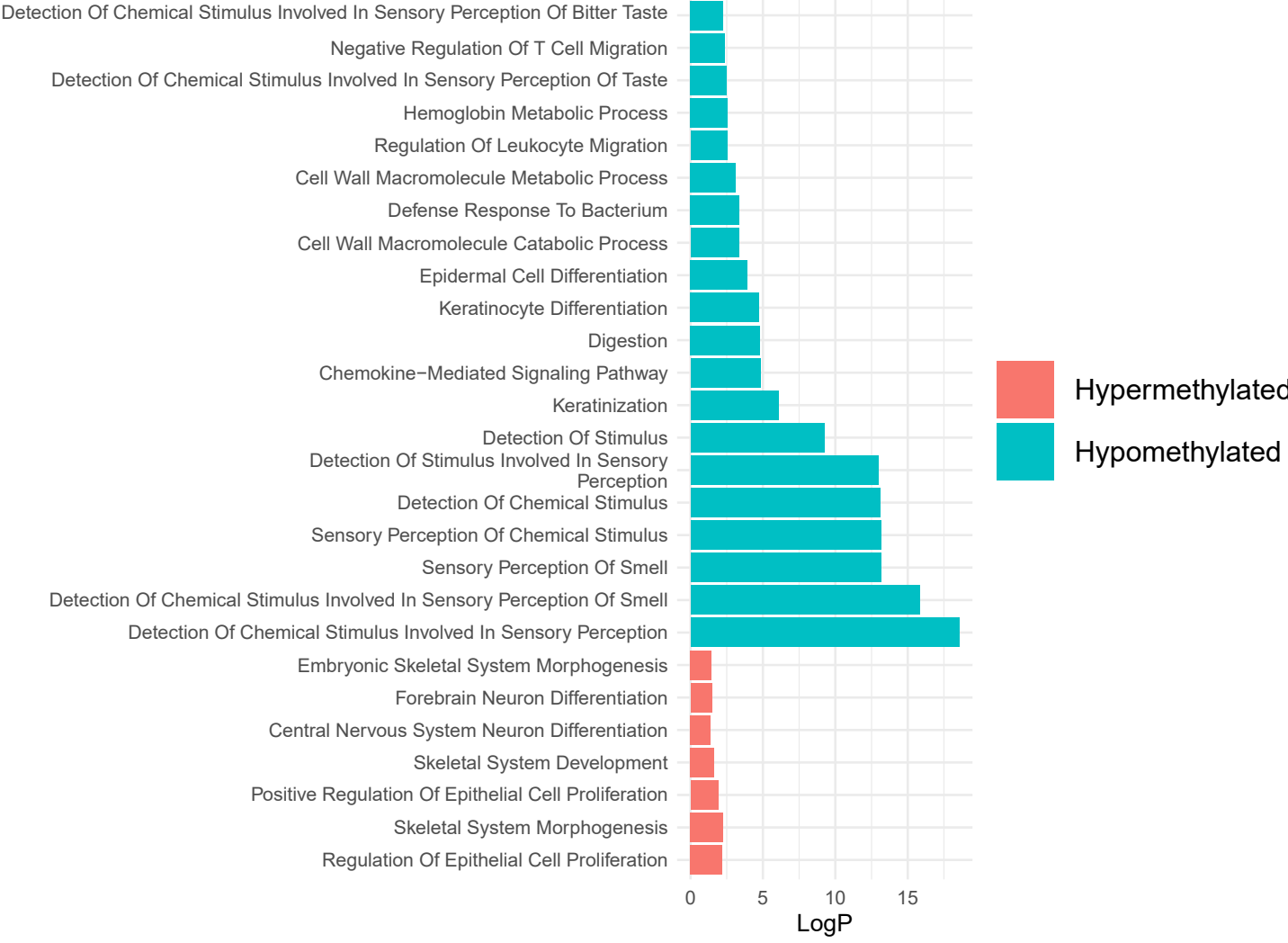

Supplement: Supplementary file 10 — Additional file 10: Suppl Figure 10. Pathways enriched in genes near hypermethylated or hypomethylated CpG sites in Classical, Mesenchymal, and Proneural subtype patients compared to the rest of patients. [file 13040_2021_273_MOESM10_ESM.pdf]
