# Supplementary material for: iGlioSub: an integrative transcriptomic and epigenomic classifier for glioblastoma molecular subtypes
Source: BioData Min. 2021 Aug 23;14:42. doi: 10.1186/s13040-021-00273-8 (PMC8381510; doi:10.1186/s13040-021-00273-8)
Supplement: Supplementary file 2 — Additional file 2: Suppl Figure 2. t-SNE plots displaying the distribution of samples coming from different DNA methylation arrays and sources, before and after correction of batch effect. The batch effect was corrected using the ComBat function in the R/sva package. [file 13040_2021_273_MOESM2_ESM.pdf]

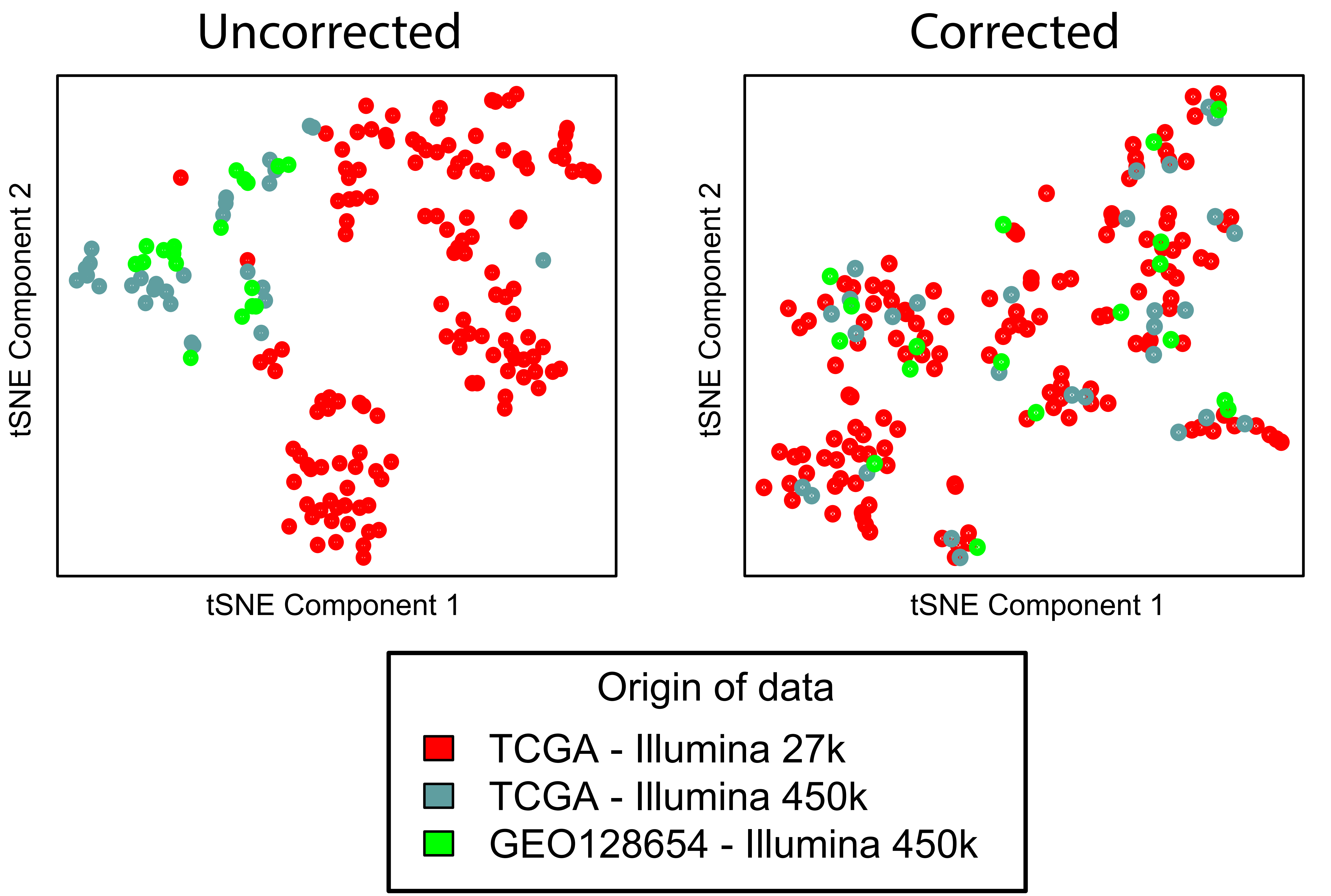
Uncorrected
Corrected
tSNE Component 2
tSNE Component 1
tSNE Component 2
tSNE Component 1
Origin of data
TCGA - Illumina 27k
TCGA - Illumina 450k
GEO128654 - Illumina 450k
